# Efficacy of nonpharmacological interventions targeting social function in children and adults with autism spectrum disorder: A systematic review and meta-analysis

**DOI:** 10.1371/journal.pone.0291720

**Published:** 2023-09-19

**Authors:** Zhili Yu, Peiming Zhang, Chenyang Tao, Liming Lu, Chunzhi Tang

**Affiliations:** Medical College of Acu-Moxi and Rehabilitation, Guangzhou University of Chinese Medicine, Guangzhou, People’s Republic of China; Ospedale Sant’Antonio, ITALY

## Abstract

**Background and aims:**

This paper aimed to evaluate the use of nonpharmacological interventions for the management of autism spectrum disorder (ASD). The effects of acupuncture and behavioural therapy, two nonpharmalogical interventions, on social function in ASD patients are still controversial. This meta-analysis investigated the impact of these two treatments and compared their effects.

**Methods:**

Seven electronic databases were systematically searched to identify randomized controlled trials (RCTs) on the use of acupuncture or behavioural therapy for ASD. A meta-analysis was carried out using Review Manager 5.4 software. Continuous data are reported as mean differences (MDs) or standardized mean differences (SMDs) with 95% confidence intervals (CIs). An assessment of methodological quality using the Cochrane risk-of-bias (ROB) tool for trials was carried out. The Grading of Recommendation Assessment, Development, and Evaluation (GRADE) was applied to evaluate the quality (certainty) of evidence for results regarding social function indicators.

**Results:**

Thirty RCTs on acupuncture and 36 on behavioural therapy were included. Compared with the control condition, body acupuncture (SMD: 0.76, 95% CI: [0.52, 1.01]; low certainty), modern acupuncture technology (SMD: 0.84, 95% CI: [0.32, 1.35]; low certainty), cognitive behavioural therapy (SMD: 0.42, 95% CI: [0.26, 0.58]; high certainty), the Denver model (SMD: 0.61, 95% CI: [0.23, 0.99]; moderate certainty) and social skills training (SMD: 0.56, 95% CI: [0.41, 0.71]; moderate certainty) improved social functioning.

**Conclusion:**

Behavioural therapies (such as CBT, the Denver model, social skills training), improved the social functioning of patients with ASD in the short and long term, as supported by high- and moderate-quality evidence. Acupuncture (including scalp acupuncture, body acupuncture and use of modern acupuncture technology) also improved social functioning, as supported by low- and very low-quality evidence. More high-quality evidence is needed to confirm the effect of acupoint catgut embedding and Early Intensive Behavioural Intervention (EIBI).

## 1 Introduction

Autism spectrum disorder (ASD) is a global health problem. In 2021, the US Centers for Disease Control (CDC) reported that among American 8-year-olds, 1 in every 44 have ASD. These statistics represent a 23% increase in prevalence over the past two years. This increased prevalence (i.e., the prevalence in 2021) is 3.39 times higher than the prevalence of autism first reported by the CDC in 2000. In China, survey data show that children with ASD (prevalence rate of 1%) account for 36.9% of children with intellectual disabilities [1]. Studies from some European countries have also shown a considerable increase in estimated ASD prevalence recently [2]. The increased prevalence among Eastern and Western countries imposes financial burdens on families and on society. For example, a 2018 Chinese survey showed that nearly 30 000 RMB was spent on children with ASD in the past 12 months [3]. Social dysfunction is one of the two leading indicators of ASD, arising from impairments in several functional domains [4]. Unfortunately, these impairments are exacerbated rather than ameliorated over development [5]. Nonpharmacological interventions are considered a safe treatment for ASD.

Nonpharmacological therapy, including behavioural therapy and acupuncture, is effective as a complementary and alternative medicine (CAM) [6–9]. The European Society for Child and Adolescent Psychiatry (ESCAP) recommends behavioural interventions for ASD that are developmentally based and involve social communication therapies and interventions based on administered behaviour evaluation, including Early Intensive Behaviour Intervention (EIBI), Naturalistic Developmental Behavioural Intervention (NDBI), the Early Start Denver Model (ESDM), social skill programs, and cognitive behavioural theory (CBT).

Most meta-analyses have examined the overall effect of acupuncture on ASD rather than specific aspects such as its effect on social function [8, 10]; hence, a systematic evaluation and quantitative analysis of clinical evidence for the use of behavioural therapy and acupuncture for ASD and their effects on social function is needed. This study aimed to identify the short- and long-term effects of acupuncture and behavioural therapy on social function among ASD patients. The benefits of behavioural therapy and acupuncture were also compared.

## 2 Methods

The review protocol was registered in the International Prospective Register of Systematic Reviews (PROSPERO) on November 3^rd^, 2021, and revised on May 26^th^, 2022 (registration number: CRD42021284512).

### 2.1 Eligibility criteria

The inclusion criteria were as follows for studies identified in the literature review: (1) included participants with a diagnosis of ASD, regardless of age, sex, or race; (2) included at least one of the eight kinds of intervention type; (3) included a control group that underwent regular psychotherapy, therapy as usual, registration on a waitlist, or no intervention; (4) included indexes of social function or outcomes of social function, assessed before and after treatment; (5) was an RCT; and (6) was published in Chinese or English. Studies were excluded if they (1) applied specific or rarely used psychotherapy in the control group or (2) failed to report sufficient data to calculate the mean difference (MD) or standardized mean difference (SMD). The eligibility criteria listed above are also displayed in Table 1.

**Table 1:** Inclusion and exclusion criteria for the studies.

	Inclusion criteria	Exclusion criteria
Population	Participants with a diagnosis of ASD, regardless of age, sex, or race	
Intervention	Included at least one of the eight kinds of intervention type (scalp acupuncture, body acupuncture, modern acupuncture technology, acupoint catgut embedding, cognitive behavioural therapy, the Denver model, social skills training, or early intensive behavioral intervention)	
Comparison	A control group that underwent regular psychotherapy, therapy as usual, registration on a waitlist, or no intervention	Applied specific or rarely used psychotherapy in the control group
Outcome	Indexes of social function or outcomes of social function, assessed before and after treatment	Failed to report sufficient data to calculate the mean difference (MD) or standardized mean difference (SMD)
Study design	RCT	
Other	Written in Chinese or English.	

### 2.2 Data sources and search strategies

The databases searched included PubMed, Embase, Web of Science, Allied and Complementary Medicine Database (AMED), PsycINFO, and CNKI and WanFang (databases of Chinese medical journals). RCTs on acupuncture and behavioural therapy were identified (details shown in the figure in S1 Material in S1 File). The literature search spanned from database inception to April 26^th^, 2022.

### 2.3 Study selection and literature search

All articles were identified and screened by two independent reviewers (Zhili Yu (ZY) and Chenyang Tao (CT)) using NoteExpress; any disagreements were resolved through discussion. If the articles were inaccessible, researchers contacted the authors to obtain them; if the authors were unavailable, the articles were excluded.

### 2.4 Data extraction

Two independent reviewers (ZY and CT) extracted the following information from the included studies: first author, publication year, country of origin, study population, age range or mean age, numbers of cases and controls, treatment methods for each group (a brief overview of the types of interventions included is shown in S2 Material Table 2 in S1 File), sample size of the study population in the last intervention, social function indicators, intervention duration, follow-up period duration and social function outcomes (mean and SD). If the indexes of social function encompassed two or more scales or two or more indexes were related to the same indicator of social function, we chose the scale or index most frequently used in other studies to control for known sources of heterogeneity.

### 2.5 Quality assessment

The risk of bias was evaluated by the Cochrane Collaboration’s risk-of-bias (ROB) tool, which assesses the risk of bias in seven aspects on three grades: low, high, and unclear risk of bias. Finally, an overall ROB rating was obtained for each included study. Disagreements over the ROB between the reviewers (ZY and CT) were resolved through discussion with another author (Peiming Zhang (PZ)). A table presenting the risk of bias was generated with Revman 5.4 software.

### 2.6 Effect measures

Pretreatment, posttreatment, and follow-up data were extracted. The meta-analysis assessed differences between pretreatment and posttreatment as well as between pretreatment and follow-up. The social function indicators included eight positive scales (the Griffith Mental Development Scale (GMDS), Functional Independence Measure for Children (WeeFIM), Pediatric Quality of Life Inventory (PedsQL), Social Life Ability Scale(SM), Social Competence with Peers Questionnaire (SCPQ), Test of Adolescent Social Skills Knowledge (TASSK), Social Support Questionnaire (SSQ), and Social Skills Rating Scale (SSRS)) and eight negative scales (the Autism Behavior Checklist (ABC), Autism Treatment Evaluation Checklist (ATEC), Childhood Autism Rating Scale (CARS), Children’s Automatic Thoughts Scale (CATS), Social Responsiveness Scale (SRS), Spence Children’s Anxiety Scale (SCAS), Vineland Adaptive Behavior Scales (VABS), and Contextual Assessment of Social Skills (CASS)). If a study failed to report all necessary data, we attempted to contact the authors of the study; if the authors were not reached, we excluded the study. Brief overviews of the scales measuring social function indicators are provided in S2 Material Table 1 in S1 File.

### 2.7 Quality of evidence

For studies included in the meta-analysis, GRADEpro GDT software (GRADEpro GDT 2022) was used to determine the quality of evidence according to the Cochrane-recommended GRADE domains: risk of bias, inconsistency, indirectness, imprecision, and publication bias [11]. If limitations were identified, the quality (certainty) of evidence was downgraded according to the guidelines.

### 2.8 Statistical analysis

The statistical analysis was performed using Review Manager 5.4 software. Changes in social functioning were computed using MD with 95% confidence intervals (CIs) if the outcome measures were the same across studies and SMDs with 95% CIs when the outcome measures differed across studies. If only baseline and final scores were reported, a formula [12] was adopted to determine the change in social function. Regarding changes in social functioning measured with negative scales (i.e., on which higher scores reflected worse social functioning), the change was multiplied by -1 to standardize the direction of effects with that of the positive scales [12]. Moreover, all outcome data were transformed to the MD and SMD as appropriate, following the Cochrane Handbook for Systematic Reviews of Interventions. The I^2^ test was used to analyse the heterogeneity of the pooled studies. Fixed-effects models were used unless I^2^>50%; in this case, a random-effects model was used, and the causes of heterogeneity were explored through subgroup or sensitivity analyses. If the quantity of included studies was adequate (n≥10), a funnel plot was constructed to assess publication bias [10].

## 3 Results

**Fig 1:**
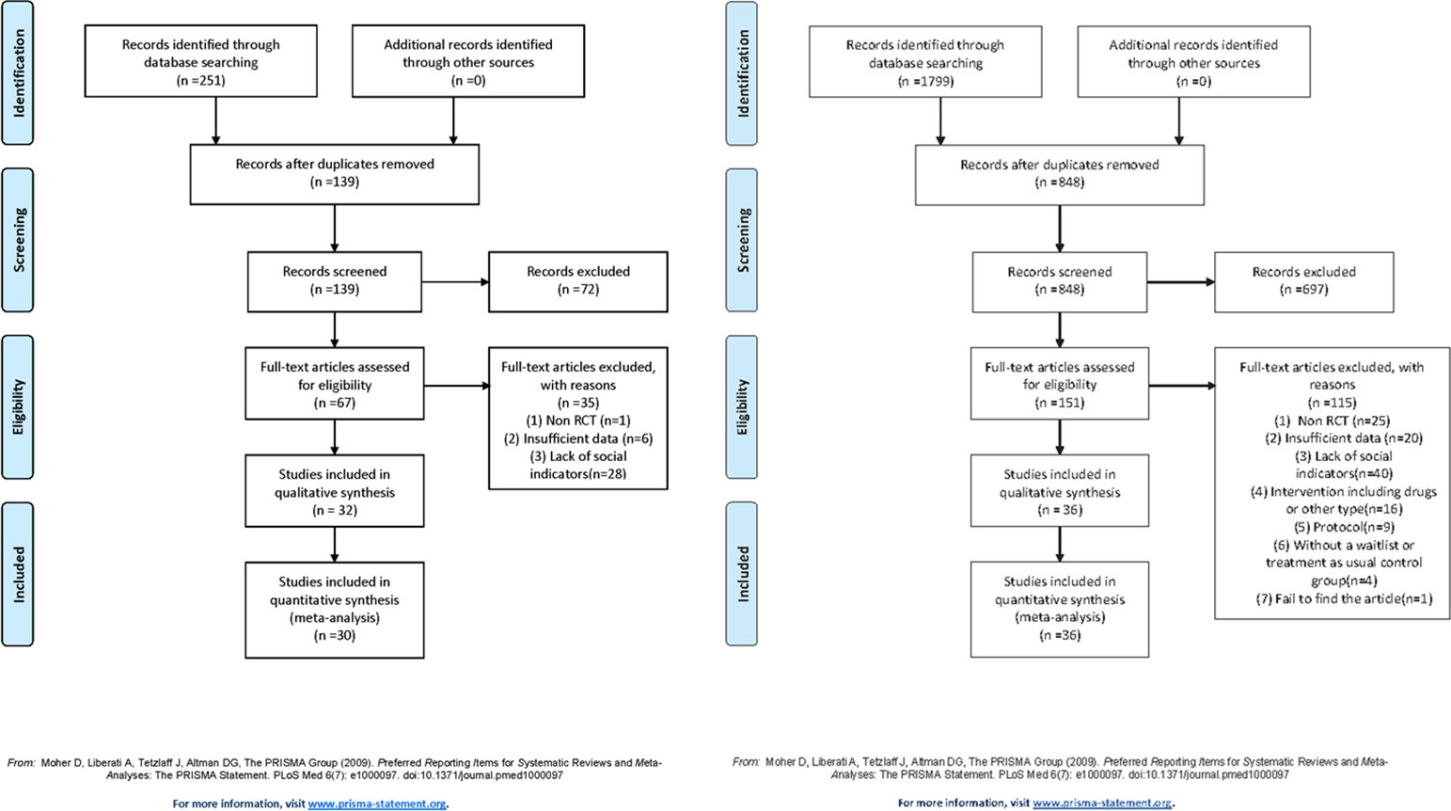
**a.** Preferred Reporting Items for Systematic Reviews and Meta-Analyses (PRISMA) flowchart of included studies on acupuncture. **b.** PRISMA flowchart of included studies on behavioural therapy.

The literature search of the seven databases initially identified 1799 behavioural therapy RCTs and 251 acupuncture RCTs. Of these, 951 behavioural therapy and 112 acupuncture RCT duplications were excluded; 697 behavioural therapy and 71 acupuncture publications were removed after review of the title and abstract, and 115 behavioural therapy and 37 acupuncture articles were excluded after review of the full text. Finally, 36 behavioural therapy and 32 acupuncture studies were included. The screening steps are displayed in Fig 1A and 1B.

All included studies were RCTs. The characteristics of the participants, interventions, controls, and outcomes are described below.

Thirty-two studies administered acupuncture plus regular psychotherapy in the treatment group (Table 2). Participant ages ranged from 1 to 18 years old. Regarding the control group, all articles administered regular psychotherapy. Only two studies used sham or nonpoint acupuncture as a control group; these studies administered electroacupuncture to the treatment group. The most common social function indicator used in acupuncture studies was the ABC. Although the intervention durations were variable among acupuncture studies, most administered 70 to 80 sessions.

**Table 2:** Summary of treatment and treatment-related information from included studies on acupuncture.

Authors, years	Age of Participants	Sample(T/C)	Intervention(C)	Intervention(T)	Social Outcome indicators	Intervention length
Yanrong, G.et al., 2021	Age 4–12 years	25/25	Analysis behavior therapy	C+ scalp acupuncture	ABC	60 sessions
Huijie, L. 2022	Age 3–7 years	31/31	Auditory integration training	C+ scalp acupuncture	ABC	36 sessions
Lianjun, L.2017	Age 2.5–6.7 years	43/40	Music therapy	C+ scalp acupuncture	Gesell	30 sessions
Yanna, H.2018	Age 1–2 years	40/40	Treatment as usual+ Jiao’s scalp needle	C+ Lin’s scalp acupuncture	Gesell	30 sessions
Lina, Y. et al.2021	Under 12 years	53/53	Treatment as usual	C+ scalp acupuncture	ABC	18 sessions
Yamei, L. et al., 2019	Age 1.3–6.6 years	35/35	Language training	C+ scalp acupuncture	ATEC	80 sessions
Ping, W.2021	Age 2–7 years	40/40	Discrete Trial Teaching	C+ scalp acupuncture	Gesell	77 sessions
Huiqin & Yushun, 2019	Age 1–11 years	40/40	Behavioral therapy	C+ scalp acupuncture	ABC	12 sessions
Jin, Y.et al.2016	Age 3–6 years	40/40	Treatment as usual	C+ scalp acupuncture	Gesell	60 sessions
Renhua, M. et al.2017	Age 1–6 years	32/32	Treatment as usual	C+ scalp acupuncture	ABC	28 sessions
Hongtao, Z. et al.2018	Age 1–11 years	45/45	Behavioral therapy	C+ scalp acupuncture	ABC	12 sessions
Nuo, L. et al., 2017	Age 2–6 years	45/45	Music therapy+ structured teaching	C+ body acupuncture	Gesell	90 sessions
Kong, F. et al., 2018	Under 14 years	30/30	Language training + behavior training + cognitive training	C+ body acupuncture	Gesell	102 sessions
Feng, G. et al., 2019	Age 2–12 years	30/30	Language training + behavior training + multi-sense training	C+ body acupuncture	ATEC	77 sessions
Yanli, W. et al., 2020	Age 2–6 years	35/35	Language training + behavior training + critical response training	C+ body acupuncture	ABC	75 sessions
Guo-Xiang, Z. et al.2020	Average age 5±1 years	55/55	Behavior analysis+ language training+ music therapy+ multi-sense training	C+ body acupuncture	CARS	90 sessions
Weili, D. et al., 2020	Age 3–6 years	43/43	Treatment as usual	C+ body acupuncture	ATEC	77 sessions
Ying, T.2022	Age 5–10 years	50/50	Treatment as usual	C+ body acupuncture	PedsQL	90 sessions
Longsheng, H. et al.2021	Age 3–7 years	25/25	Applied behavioral analysis + TEACCH	C+ needle-embedding therapy+ Jin’s three-needle therapy	ABC+CARS	60 sessions
Lan, R. et al., 2021	Age 2–8 years	54/54	Treatment as usual	C+ body acupuncture	ABC	80 sessions
Jingang, Z.2021	Age 2–9 years	44/44	Interest-based floor play therapy	C+ body acupuncture	ABC	60 sessions
Caixia & Yongchao 2020	Age 1–7 years	25/25	Treatment as usual	C+ body acupuncture	SM	28 sessions
Wen & Xiangdong, 2020	Control: 2–9 years; Treatment: 22–41 years	35/35	Structured teaching pattern	C+ body acupuncture	ABC	72 sessions
Bing-xu, J. et al.2020	Age 3–6 years	30/30	Conductive education+ language training+ music therapy	C+ acupoint catgut embedding	ABC	15 sessions
Wen-Liu, Z. et al.2021	Age 3–6 years	30/30	Language training+ applied behavior analysis+ multi-sense training	C+ acupoint catgut embedding	Gesell	18 sessions
				C+ scalp acupuncture	Gesell	128 sessions
Wong & Chen2010	Age 3–18 years	30/25	Non-point electroacupuncture+ treatment as usual	Electroacupuncture+ treatment as usual	WeeFIM	14 sessions
Wang, C. N. et al.2007	Age 3–9 years	30/30	Behavioral therapy	C+ Electroacupuncture	ABC	80 sessions
Surapaty, I. A. et al.2020	Age 2–6 years	23/23	sensory–occupational integrative therapy+placebo acupuncture	sensory–occupational integrative therapy+verum laser acupuncture	WeeFIM	18 sessions
Wanqiong, Z.2022	Age 5–11 years	35/35	Treatment as usual	C+ Electroacupuncture	ABC	72 sessions
Ningxia, Z.et al., 2015	Age 2.5–6 years	30/30	Treatment as usual	C+ Electroacupuncture	ABC	72 sessions
Nuo, L. et al., 2011	Age 2–6 years	30/40	Music therapy+ conductive education	C+ scalp acupuncture	Gesell	60 sessions
Yong, Z. et al., 2015	Age 1–2 years	33/32	Treatment as usual+Jiao scalp needle	C+ Lin scalp acupuncture	Gesell	45 sessions

*ABC: Autism Behavior Checklist; ATEC: Autism Treatment Evaluation Checklist; CARS: Childhood Autism Rating Scale; PedsQL: Pediatric Quality of Life Inventory; SM: social life ability scale; WeeFIM: Functional Independence Measure for Children

Thirty-six studies administered behavioural therapy (Table 3). Participant age ranged from 1.5 to 65 years old. The control group in behavioural therapy studies most frequently involved a waitlist control. The SRS was the most commonly used social function indicator. The intervention durations less variable among behavioural therapy studies than acupuncture studies; most studies administered 14 to 16 sessions. Only four studies provided follow-up results after treatment, and all administered behavioural therapy. The length of the follow-up period ranged from 6 weeks to 2 years.

**Table 3:** Summary of treatment and treatment-related information from included studies on behavioural therapy.

Authors, years	Age of Participants	Sample(T/C)	Intervention(C)	Intervention(T)	Social Outcome indicators	Intervention length	Length of follow-up
Andrews, L. et al., 2013	Age 7–12 years	29/29	Waitlist control	CBT	SCPQ	Ten sessions	
Langdon, P. E. et al.2016	Age 17–65 years	23/23	Waitlist control	CBT	Social Phobia Inventory	24 sessions	
Kilburn, T. R. et al., 2020	Age 8–14 years	25/24	Waitlist control	the Cool Kids ASD program	CATS	Ten sessions	
Koning, C. et al.2013	Age 10–12 years	7/8	Waitlist control	CBT-based social skills intervention	SRS	15 sessions	
Sofronoff, K. et al.2005	Age 10–12 years	21/23	Waitlist control	CBT (children only)	SCAS-P	Six sessions	Six weeks
				CBT (child + parents)			
Freitag,C.M.et al.2016	Age 8–20 years	101/108	Treatment as usual	SOSTA-FRA	SRS	12 sessions	Three months
Ireri, N. W. et al.2019	Age 5–24 years	20/20	Waitlist control	MASSI	SRS	20 sessions	
Wood, J. J. et al., 2015	Age 11–15 years	19/14	Waitlist control	BIACA	SRS	16 sessions	
Wood, J. J. et al., 2009	Age 7–11 years	9/10	Waitlist control	CBT	SRS	16 sessions	
Storch, E. A. et al.2015	Age 11–16 years	16/15	Treatment as usual	BIACA	SRS	16 sessions	
Storch, E. A. et al.2013	Age 7–11 years	22/21	Treatment as usual	CBT	SRS	16 sessions	
Dawson, G. et al.2010	Age 1.5–2.5 years	24/24	the assess-and-monitor	ESDM	VANS	1042 sessions	
Hong-Hua, L. et al.2018	Age 2–5 years	17/18	Treatment as usual	ESDM	ABC	77 sessions	
Rogers, S. J. et al.2012	Age 1–2 years	49/49	Treatment as usual	ESDM	VANS	12 sessions	
Shihuan, W. et al.2021	Age 1–3 years	23/26	Waitlist control	ESDM	Gesell	24 sessions	
Gantman, A. et al.2012	Age 1.5–2 years	9/8	Waitlist control	The UCLA PEERS	SRS	14 sessions	
Vernon, T. W. et al.2019	Age 1.5–4.7 years	12/11	Waitlist control	the Pivotal Response Intervention for Social Motivation	VANS	26 sessions	
Leaf, J. B. et al.2017	Age 3–7 years	8/7	Waitlist control	Social skills groups	SRS	32 sessions	
McVey, A. J. et al.2016	Age 18–28 years	24/23	Waitlist control	PEERS	SRS	14 sessions	
Dolan, B. K. et al.2016	Age 11–16 years	28/25	Waitlist control	PEERS	CASS	14 sessions	
Thomeer, M. L. et al. 2012	Age 7–12 years	17/17	Waitlist control	a comprehensive psychosocial intervention	SRS	Six sessions	
Yoo, H. et al.2014	Age 12–18 years	23/24	Waitlist control	Korean PEERS® Treatment	TASK	14 sessions	
Laugeson, E. A. et al.2015	Age 18–24 years	9/8	Waitlist control	PEERS	SRS	16 sessions	
Temkin, A. B. et al.2022	Age 8–12 years	42/40	Treatment as usual	Secret Agent Society	SSQ	Nine sessions	
Rabin, S. J. et al.2021	Age 12–17 years	36/35	Waitlist control	PEERS	SRS	16 sessions	
White, S. W. et al., 2013	Age 12–17 years	15/15	Waitlist control	MASSI	SRS	20 sessions	
Gorenstein, M. et al.2020	Age 18–45 years	11/11	Waitlist control	Job-Based Social Skills Program	SRS	15 sessions	
Kazemi & Abolghasemi 2019	Average age 11.88±2.29 years	4/4	No intervention	Play-based empathy training	SRS	18 sessions	
Dekker, V. et al.2019	Age 9.6–13 years	26/26	Treatment as usual	Social Skills Training	SSRS	15 sessions	
				Social Skills Training- parent and teacher involvement			
De Korte, M.W.P.et al.2021	Age 9–15 years	17/19	Treatment as usual	Pivotal Response Treatment	SRS	12–20 sessions	Eight weeks
Chien, Y. L. et al.2021	Age 18–45 years	36/36	Treatment as usual	PEERS	SRS	16 sessions	
Bauminger-Zviely, N. et al.2020	Age 8–16 years	18/18	Treatment as usual	Conversation	VANS	60 sessions	
		18		Collaboration			
Barrett, A. C.et al.2020	Age 1.5–4.5 years	12/9	Waitlist control	Pivotal Response Treatment	Parent-child play interaction videos	6.81h/week, six months total	
Adibsereshki, N. et al.2015	Age 7–12 years	12/12	Regular school program	Theory of Mind	SSRS	15 sessions	
Kovshoff, H et al.2011	Age 6.5–8 years	23/18	Treatment as usual	EIBI	VANS	Two years	Two years
Whitehouse, A.et al., 2017	Average age: Control 40.25±8.41 months; Treatment 39.36±8.50 months	36/35	Treatment as usual	C+ TOBY(app based an app-based learning curriculum)	VANS	180 sessions	

*CBT: cognitive behavioural therapy; SCPQ: Social Competence with Peers Questionnaire; CATS: Children’s Automatic Thoughts Scale; SRS: Social Responsiveness Scale; SCAS: Spence Children’s Anxiety Scale; SOSTA-FRA: Social Skills Training Autism–Frankfurt; MASSI: Multimodal Anxiety and Social Skills Intervention; BIACA: Behavioural Interventions for Anxiety in Children with Autism; ESDM: Early Start Denver Model; VABS: Vinland Adaptive Behavior Scales; UCLA PEERS: University of California at Los Angeles Program for the Education and Endowment of Relational Expertise; CASS: Contextual Assessment of Social Skills; TASSK: Test of Adolescent Social Skills Knowledge; SSQ: Social Skill Questionnaire; SSRS: Social Skills Rating Scale.

**Fig 2:**
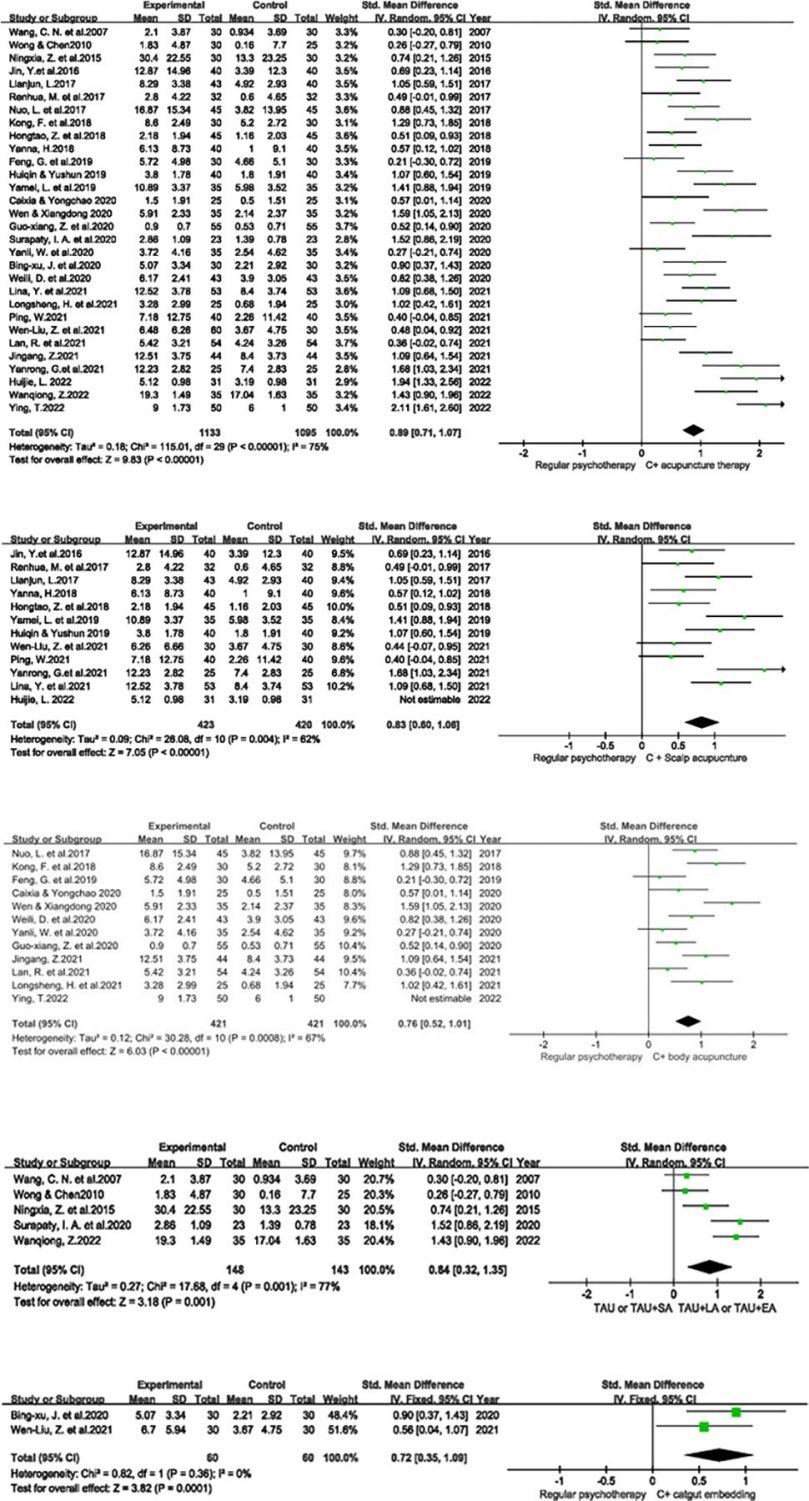
**a** Forest plot of the overall effect of acupuncture. *C: Control. **b** Forest plot of the effect of scalp acupuncture**. c** Forest plot of body acupuncture. **d** Forest plot of the effect of modern acupuncture technology. *TAU: treatment as usual; EA: electroacupuncture; LA: laser acupuncture; SA: sham acupuncture. **e** Forest plot of the effect of acupoint catgut embedding.

**Fig 3:**
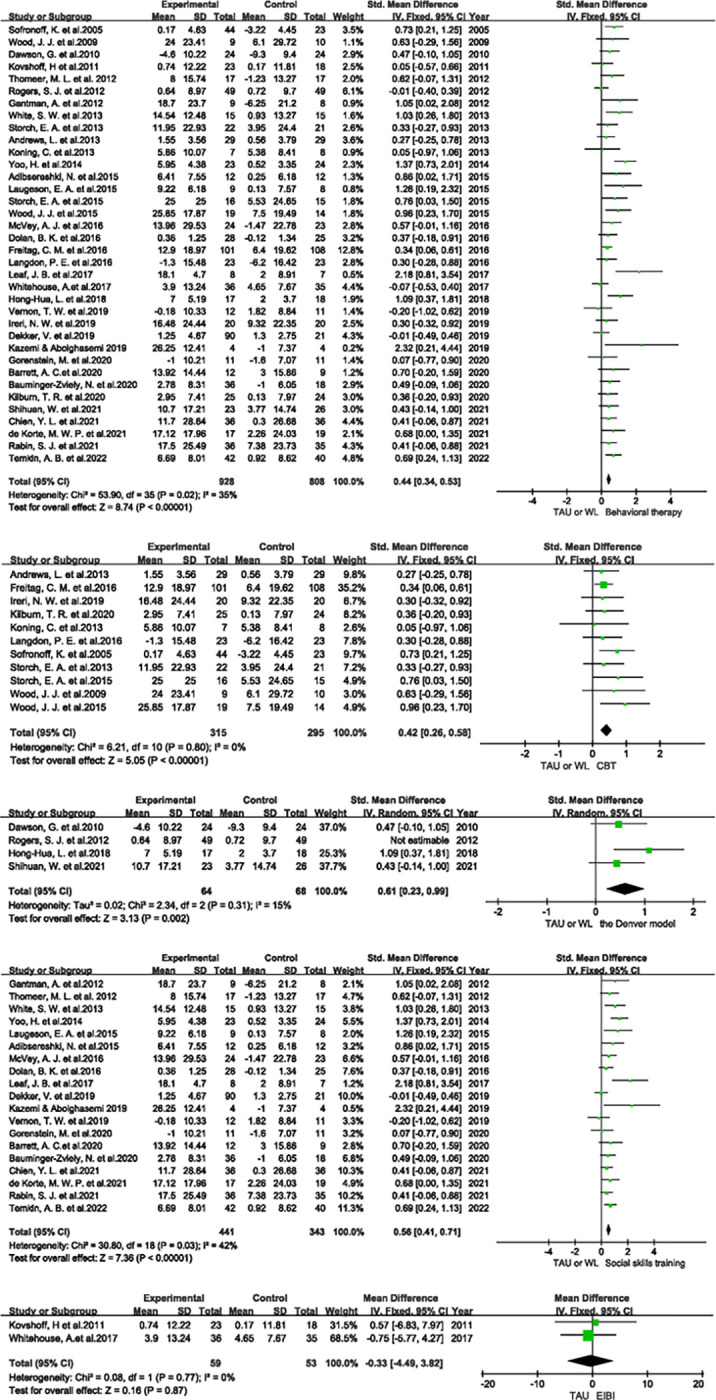
**a** Forest plot of the overall effect of behavioural therapy. *WL: Waitlist. **b** Forest plot of the effect of cognitive behavioural therapy. **c** Forest plot of the effect of the Denver model. **d** Forest plot of the effect of social skills training. **e** Forest plot of the effect of Early Intensive Behavioural Intervention.

The overall risk of bias of the included studies followed the suggested framework and was categorized as follows: high risk (2.90%), unclear risk (75.00%), and low risk (22.06%) (S3 Material Table 1 and Fig 3 in S1 File). More specifically, regarding random sequence generation, 32 studies were rated as "low risk”, and two studies were rated as "high risk”. Regarding allocation concealment, 14 studies were rated as "low risk”. Regarding the blinding of participants and personnel, two studies were rated as "low risk", and the other 66 were rated as "high risk”. Regarding blinding of outcome assessment, 17 studies were rated as “low risk”. All studies were rated as “unclear risk" regarding incomplete outcome data. Regarding other biases, one study was rated as "high risk", and the other 68 studies were rated as "low risk" (details are provided in S3 Material Figs 1 and 2 in S1 File). Specific sources of risk are outlined in S3 Material Table 2 in S1 File.

The evaluation of the overall effect of acupuncture included 30 studies; overall, acupuncture improved social function to a greater extent than the control condition (SMD: 0.89, 95% CI: 0.71, 1.07). A random-effects model was applied for analysis given the considerable heterogeneity (*I*^*2*^ = 75%); a significant difference was observed (*P*<0.00001) (Fig 2A).

Analysis of the effect of scalp acupuncture included eleven studies; scalp acupuncture improved social function to a greater extent than the control condition (SMD: 0.83, 95% CI: 0.60, 1.06). A random-effects model was applied given the moderate heterogeneity (*I*^*2*^ = 62%); a significant difference was observed (*P*<0.00001) (Fig 2B).

Analysis of the effect of body acupuncture included eleven studies; body acupuncture improved social function to a greater extent than the control condition (SMD: 0.76, 95% CI: 0.52, 1.01). A random-effects model was applied given the moderate heterogeneity (*I*^*2*^ = 67%); a significant difference was observed (*P*<0.00001) (Fig 2C).

Analysis of the effect of modern acupuncture technology included five studies; modern acupuncture technology improved social function to a greater extent than the control condition (SMD: 0.84, 95% CI: 0.32, 1.35). A random-effects model was applied given the considerable heterogeneity (*I*^*2*^ = 77%); a significant difference was observed (*P* = 0.001) (Fig 2D).

Analysis of the effect of acupoint catgut embedding included two studies; acupoint catgut embedding improved social function to a greater extent than the control condition (SMD: 0.72, 95% CI: 0.35, 1.09). As there was no heterogeneity (*I*^*2*^ = 0%), a fixed-effects model was applied, and a significant difference was observed (*P* = 0.0001) (Fig 2E).

Analysis of the overall effect of behavioural therapy included 36 studies; behavioural therapy improved social function to a greater extent than the control condition (SMD: 0.44, 95% CI: 0.34, 0.53). A fixed-effects model was applied as there was little heterogeneity (*I*^*2*^ = 35%), and a significant difference was observed (*P*<0.00001) (Fig 3A).

Analysis of the effect of cognitive behavioural therapy (CBT) included eleven studies; CBT improved social function to a greater extent than the control condition (SMD: 0. 42, 95% CI: 0.26, 0.58). There was no heterogeneity (*I*^*2*^ = 0%); thus, a fixed-effects model was applied, and a significant difference was observed (*P*<0.00001) (Fig 3B).

Among CBT studies, Langdon, P. E. et al. (2016) evaluated social functioning with the Social Phobia Inventory (SPIN), in which the variables reflect adverseness to the participants. As a result, SPIN scores were multiplied by -1 during meta-analysis. Sofronoff, K. et al. (2005) assessed social functioning with the parent-report version of the SCAS (SCAS-P); changes in SCAS-P scores were multiplied by -1 for the same reason. Furthermore, this RCT was a 3-arm experiment, in which the treatment group was divided into CBT (children only) and CBT (children + parents); thus, the data were combined using the same formula mentioned above.

Analysis of the effect of the Denver model included three studies; the Denver model improved social function to a greater extent than the control condition (SMD: 0.61, 95% CI: 0.23, 0.99). There was little heterogeneity (*I*^*2*^ = 15%); thus, a fixed-effects model was applied, and a significant difference was observed (*P* = 0.002) (Fig 3C).

Dawson, G. et al. (2010) evaluated social outcomes with the VABS and showed declines in the treatment and control groups. As higher VBAS scores reflect worse social functioning, we multiplied these scores by -1.

Analysis of social skills training included 19 studies; social skills training improved social function to a greater extent than the control condition (SMD: 0.56, 95% CI: 0.41, 0.71). There was little heterogeneity (*I*^*2*^ = 42%); thus, a fixed-effects model was applied, and a significant difference was observed (*P*<0.00001) (Fig 3D).

Seven studies assessed social functioning with the SRS, CASS, or VABS; higher scores on these scales reflects worse social functioning. Thus, we multiplied these scores by -1 during analysis.

Analysis of the effect of EIBI included two studies; EIBI did not improve social functioning to a greater extent than the control condition (MD -0.33, 95% CI: -4.49, 3.82). There was no heterogeneity (*I*^2^ = 0%); thus, a fixed-effects model was applied, but no significant difference was observed (*P* = 0.87) (Fig 3E).

**Fig 4:**
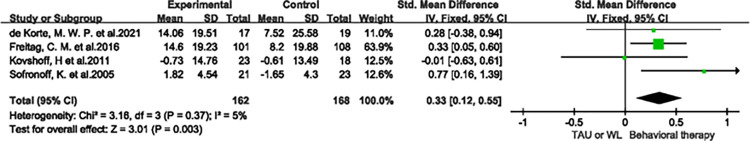
Forest plot of the long-term effect of behavioural therapy.

Analysis of the long-term effect of behavioural therapy included four studies; long-term behavioural therapy improved social function to a greater extent than the control condition (SMD: 0.33, 95% CI: 0.12, 0.55). There was little heterogeneity (*I*^*2*^ = 5%); thus, a fixed-effects model was applied, and a significant difference was observed (*P* = 0.003) (Fig 4).

Kovshoff, H et al. (2011) and Sofronoff, K. et al. (2005) (in a control group) reported detrimental increases in scores after treatment; therefore, we multiplied these scores by -1 during analysis.

Nearly all studies compared acupuncture plus psychotherapy with regular psychotherapy. In other words, they adopted acupuncture as a complementary therapy. An insufficient number of studies compared the effects of acupuncture and behavioural treatment directly; thus, no analysis was conducted.

Next, the source of heterogeneity was investigated. Five studies were removed. Specifically, comparison of the effects of scalp acupuncture plus psychotherapy with those of regular psychotherapy involved the initial exclusion of two high-risk studies. Subsequently, another study was excluded, which decreased *I*^*2*^ by 10%. One study was excluded when comparing the effects of body acupuncture plus psychotherapy with those of regular psychotherapy; *I*^*2*^ decreased by 14%. In the comparison of the effects of the Denver model and those of the control condition, 1 study was excluded, and *I*^*2*^ decreased by 45%.

**Fig 5:**
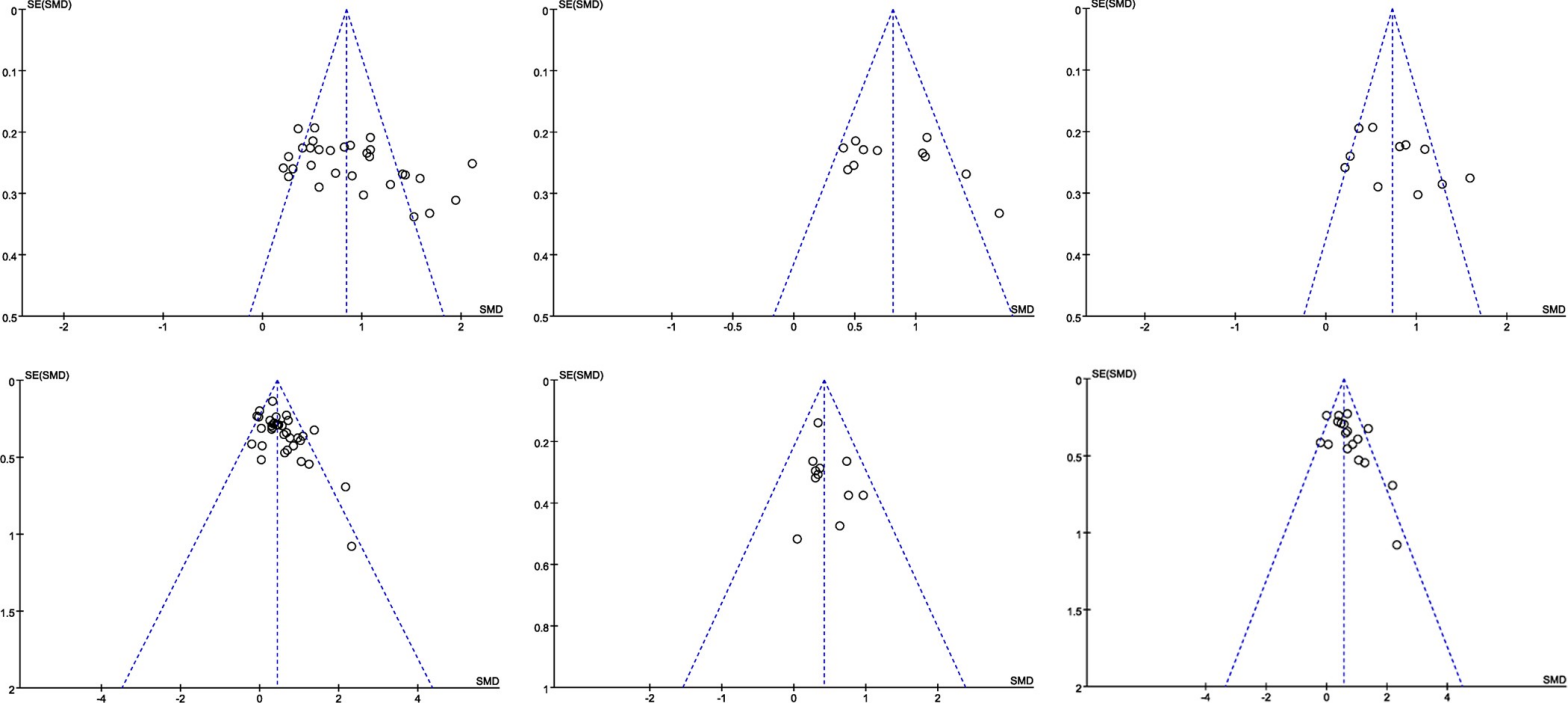
**a** Funnel plot of all acupuncture studies. **b** Funnel plot of scalp acupuncture studies. **c** Funnel plot of body acupuncture studies. **d** Funnel plot of all behavioural therapy studies. **e** Funnel plot of cognitive behavioural therapy studies. **f** Funnel plot of social skills training studies.

Regarding the types of intervention included, more than 10 studies applied acupuncture, behavioural therapy, scalp acupuncture, body acupuncture, CBT, and social skills training; the other interventions were applied in fewer than 10 studies. Funnel plot examination was performed for the six intervention types listed above. The funnel plots of studies on acupuncture, scalp acupuncture, body acupuncture, CBT, and social skills training showed some asymmetry, suggesting the presence of publication bias. (Fig 5A–5F).

The overall certainty of evidence for the effects of acupuncture was very low. Among the types of acupuncture, the quality of evidence for the effects of scalp acupuncture was very low, that for body acupuncture and modern acupuncture technology was low, and that for acupoint catgut embedding was moderate. The overall certainty of evidence for the effects of behavioural therapy was moderate. Among the types of behavioural therapy, the quality of evidence for the effect of CBT was high, that for the Denver model and social skill intervention was moderate, and that for EIBI was low. The quality of evidence for the long-term effect of behavioural therapy was moderate (details are provided in S4 Material Table 1 in S1 File).

## 4 Discussion

This systematic review and meta-analysis summarized the effects of acupuncture [13–44] and behavioural therapy [45–80] on social functioning in patients with ASD in a total of 68 RCTs. The following methods improved social functioning and had moderate- or high-quality evidence: acupoint catgut embedding (moderate certainty), behavioural therapy (moderate certainty), CBT (high certainty), the Denver model (moderate certainty), social skills training (moderate certainty) and the long-term effect of behavioural therapy (moderate certainty). Among them, acupoint catgut embedding (SMD: 0.72, 95% CI: 0.35, 1.09) significantly improved scores on social function indicators. In contrast, behavioural therapy (SMD: 0.44, 95% CI: 0.34, 0.53), CBT (SMD: 0.42, 95% CI: 0.26, 0.58), the Denver model (SMD: 0.61, 95% CI: 0.23, 0.99), social skills training (SMD: 0.56, 95% CI: 0.41, 0.71) and long-term behavioural therapy (SMD: 0.33, 95% CI: 0.12, 0.55) had moderate effects on social functioning. Although modern acupuncture technology and body acupuncture had low-quality evidence regarding improvements in social functioning, they both resulted in significant improvements in social functioning (modern acupuncture technology, SMD: 0.72, 95% CI: 0.35, 1.09; body acupuncture, SMD: 0.76, 95% CI: 0.52, 1.01).

In the investigation of the source of heterogeneity, 5 studies [13, 22, 24, 26, 57] were excluded. Two of these studies [22, 24] were rated as “high risk” for both random sequence generation and overall risk of bias [11]. The other three studies [13, 26, 57] were identified as possible sources of heterogeneity through sensitivity analysis. The potential causes of heterogeneity in these studies are as follows. Huijie, L. (2022) [13] used a different control group (auditory integration training) than the other included studies on scalp acupuncture. Ying, T. (2022) [26] used adjunct acupoints based on syndrome differentiation rather than primary acupoints plus adjunct acupoints for acupuncture, in contrast to other included studies on body acupuncture. Moreover, the authors adopted PedsQL scores as a social function indicator; this also differs from related studies. Rogers, S. J. et al. (2012) [57] had the shortest intervention duration among the 4 ESDM studies. Additionally, they did not observe a significant difference between the two groups, in contrast to the other three ESDM studies. Finally, heterogeneity in acupuncture studies remained after sensitivity analysis, but the potential cause was not identified in subgroup analysis. The possible reasons are as follows. The sample sizes of the included acupuncture studies were smaller than those in some of the behavioural studies. Since most acupuncture methods follow the theory of traditional Chinese medicine (TCM), which emphasizes individualized treatment, the selection of acupoints and needle manipulation may have varied, inducing variation in the reported results.

Compared to similar previous meta-analyses, the present has some advantages regarding improved classification methods and inclusion of a greater variety of intervention types. Among meta-analyses on the effect of acupuncture on ASD, acupuncture effects were usually categorized according to scales; little attention was given to the overall effect of acupuncture [8, 10]. Our meta-analysis concentrated on effects on social functioning and categorized acupuncture studies according to the intervention types. Regarding the effect of behavioural interventions on ASD, our meta-analysis included a wider range of behavioural therapy techniques than previous meta-analyses on social skills training [81] and focused on the effects on social functioning, which few previous CBT, Denver model, or EIBI meta-analyses examined [82–84].

This systematic review and meta-analysis has the following practical implications. (1) The effect of behavioural therapy (except EIBI) was moderate, and the long-term effect of behavioural therapy was small. Quality of evidence for these two effects were moderate and high, respectively. The former result is similar that of other meta-analyses, including one on children and adolescents with a variety of neurodevelopmental disorders that was published in *JAMA* the year before last [85]. Our meta-analysis more definitely indicates that ASD patients with social deficits could benefit from behavioural therapy. When ASD patients show signs of social impairment, behavioural therapy should be considered during the development of the clinical treatment strategy. (2) There was a significant effect of acupuncture and of the four acupuncture types when applied as adjuvant therapy (CAM). Additionally, in light of American Psychological Association (APA) guidance and a previous acupuncture meta-analysis, acupuncture as CAM may be added when the main symptom of ASD patients is social impairment. (3) The quality of evidence of most effects of acupuncture was low or very low. More rigorously designed RCTs are needed to elucidate the effects of acupuncture on social functioning in ASD patients. (4) The vast majority of acupuncture studies examined the overall effect on ASD rather than the effect on specific aspects such as social function or communication. Developing specific acupuncture points to treat specific symptoms of ASD may be a promising research direction.

The strengths of this study are as follows. (1) This meta-analysis included both acupuncture and psychotherapy studies, which are critical components of nonpharmacological interventions for ASD. (2) This study focused on social functioning in ASD patients, and social function-related data were classified and included in subgroup analysis according to the detailed intervention type. Therefore, the results of this study provide some support for the formulation of treatment plans. (3) This study evaluated the long-term effect of behavioural therapy, supporting early interventions for ASD. (4) Regarding the statistical methodology, the pretreatment-posttreatment difference was used to minimize the effect of baseline data on the results. Moreover, social function-related scales were included when possible and combined with the SMD. However, this study also has some limitations. (1) Given limited time and human resources, we focused on only the effect of the eight intervention types on social functioning in ASD according to studies published in Chinese or English. (2) Few studies applying acupoint catgut embedding or EIBI met the inclusion criteria; thus, it was difficult to evaluate the effect of the two treatments on social functioning among patients with ASD. (3) There were insufficient eligible studies to compare the effects of acupuncture and behavioural therapy on social functioning. Further advanced and multidimensional studies or more in-depth analyses, such as network meta-analyses, are needed to explore the efficacy of these two critical nonpharmacological interventions and to determine differences in the effectiveness of these two intervention types for improving social functioning.

## 5 Conclusion

Behavioural therapy, including CBT, the Denver model, social skills training and long-term behavioural therapy, enhanced social functioning in patients with ASD, as supported by high- and moderate-quality evidence. Acupuncture, including scalp, body, and modern acupuncture technology, also improved social functioning in patients with ASD, as supported by very low- and low-quality evidence. More high-quality evidence is needed to confirm the effect of acupoint catgut embedding and EIBI on social functioning in ASD.

## Supporting information

S1 File Supplementary material. (DOCX)

## References

[1] Report on the development of autism education and rehabilitation industry in China.: Beijing Normal University Publishing Group; 2022.

[2] Chiarotti F, Venerosi A. Epidemiology of Autism Spectrum Disorders: A Review of Worldwide Prevalence Estimates Since 2014. Brain Sci. 2020;10(5). doi: 10.3390/brainsci10050274 32370097 PMC7288022

[3] Zhou W, Wu K, Chen S, Liu D, Xu H, Xiong X. Effect of Time Interval From Diagnosis to Treatment on Economic Burden in Families of Children With Autism Spectrum Disorder. Front Psychiatry. 2021;12. doi: 10.3389/fpsyt.2021.679542 34899407 PMC8662780

[4] Mukherjee SB. Identification, Evaluation, and Management of Children With Autism Spectrum Disorder: American Academy of Pediatrics 2020 Clinical Guidelines. Indian Pediatr. 2020;57(10):959–62. 10.1007/s13312-020-2003-7 33089812

[5] Picci G, Scherf KS. A Two-Hit Model of Autism: Adolescence as the Second Hit. Los Angeles, CA: SAGE Publications; 2015. p. 349–71. 10.1177/2167702614540646 PMC4655890 26609500

[6] Hyman SL, Levy SE, Myers SM. Identification, Evaluation, and Management of Children With Autism Spectrum Disorder. Ameican Academy of Pediatrics. 2020. 10.1542/peds.2019-3447 31843864

[7] Fuentes J, Hervás A, Howlin P, ESCAP AWP. ESCAP practice guidance for autism: a summary of evidence-based recommendations for diagnosis and treatment. Eur Child Adoles Psy. 2020;30(6):961–84. doi: 10.1007/s00787-020-01587-4 32666205 PMC8140956

[8] Lee B, Lee J, Cheon J, Sung H, Cho S, Chang GT. The Efficacy and Safety of Acupuncture for the Treatment of Children with Autism Spectrum Disorder: A Systematic Review and Meta-Analysis. Evid-Based Compl Alt. 2018;2018:1–21. doi: 10.1155/2018/1057539 29552077 PMC5820575

[9] Jia YN, Gu JH, Wei QL, Jing YZ, Gan XY, Du XZ. [Effect of scalp acupuncture stimulation on mood and sleep in children with autism spectrum disorder]. Zhen Ci Yan Jiu. 2021;46(11):948–52. doi: 10.13702/j.1000-0607.20210276 34865332

[10] Wang L, Peng J, Qiao F, Cheng W, Lin G, Zhang Y, et al. Clinical Randomized Controlled Study of Acupuncture Treatment on Children with Autism Spectrum Disorder (ASD): A Systematic Review and Meta-Analysis. Evid-Based Compl Alt. 2021;2021:1–16. doi: 10.1155/2021/5549849 34349825 PMC8328702

[11] Cochrane Handbook for Systematic Reviews of Interventions | Cochrane Training.; 2022.

[12] Higgins J, Thomas J. the Cochrane Handbook for Systematic Reviews of Interventions.: Wiley; 2022.

[13] Huijie L. Observation on the curative effect of transhead acupuncture combined with visual and auditory training on autism. Journal of Practical Traditional Chinese Medicine. 2022;38(03):488–9.

[14] Lina Y, Chunyan Z, Yu L, Jianrong P. Study on the Effect of Scalp Acupuncture Combined with Behavioral Therapyin Children with Autism. Chinese Journal of Social Medicine. 2021;38(02):236–9.

[15] Yamei L, Yingying M, Jiajia Z, Zhe Z. Clinical observation of scalp acupuncture combined with speech training on speech rehabilitation of autistic children. Journal of Pediatrics of Traditional Chinese Medicine. 2019;15(03):71–4. 10.16840/j.issn1673-4297.2019.03.23

[16] Huiqin H, Yushun Z. Clinical Observation on 40 Cases of Infantile Autism Treated by Scalp Acupuncture Combined with Behavioral Therapy. Chinese Journal of Ethnomedicine and Ethnopharmacy. 2019;28(05):89–91.

[17] Jin Y, Xusheng G, Hongmei W, Qingrui Z. 40 cases of children with autism treated by head acupuncture combined with parent-child games and rehabilitation training. Traditional Chinese Medicinal Research. 2016;29(02):54–6.

[18] Yanrong G, Haoqiang Z, Xiaodi Z. Effect of scalp acupuncture combined with behavioral analysis therapy on joint attention and social communication ability of children with autism. World Latest Medicine. 2021;21(40):101–3. 10.3969/j.issn.1671-3141.2021.40.048

[19] Renhua M, Shanshan X. Observation on curative effect of acupuncture and moxibustion combined with comprehensive rehabilitation training on children with autism. Practical Clinical Journal of Integrated Traditional Chinese and Western Medicine. 2017;17(12):132–3. 10.13638/j.issn.1671-4040.2017.12.083

[20] Hongtao Z, Zhixiong L, Jinhua H, Yujuan X. Effects of scalp acupuncture combined with behavioral therapy on therapeutic effect, psychology and quality of life of parents in children with autism. Maternal and Child Health Care of China. 2018;33(15):3451–4. 10.7620/zgfybj.j.issn.1001-4411.2018.15.28

[21] Lianjun L. Clinical Study on Interactive Scalp Acupuncture Therapy for Autism Spectrum Disorders. Shanghai Journal of Acupuncture and Moxibustion. 2017;36(11):1303–6.

[22] Yong Z, Bingxu J, Zhenhuan L. Clinical Study on Needling LIN’s Three Temporal Acupoints for Children with Autism. Shanghai Journal of Acupuncture and Moxibustion. 2015;34(08):754–7.

[23] Yanna H. Therapeutic effect of scalp acupuncture on children with autism. Journal of Practical Traditional Chinese Medicine. 2018;34(07):833–4.

[24] Nuo L, Bingxu J, Jieling L, Zhenhuan L. Scalp acupuncture therapy for autism. Chinese Acupuncture & Moxibustion. 2011;31(08):692–6.21894689

[25] Ping W. Observation on the effect of Xingnao Kaiqiao scalp acupuncture therapy on children with autism. Journal of Frontiers of Medicine. 2021;11:182–3.

[26] Ying T. Efficacy Observation of Acupuncture Combined with Rehabilitation Training for Autism Spectrum Disorder. Shanghai Journal of Acupuncture and Moxibustion. 2022;41(04):387–91. 10.13460/j.issn.1005-0957.2022.04.0387

[27] Longsheng H, Yu H, Pin G, Ping O, Wanyu Z, Jingrong W, et al. Curative effect of Jin’s three needles therapy on core symptoms and sleep disorder in children with mild 0 to 0 moderate autism spectrum disorder. Lishizhen Medicine and Materia Medica Research. 2021;32(10):2447–50.

[28] Lan R, Jiao-wei GU, Yong W, Chen C. Effects of acupuncture combined with rehabilitation training on language communication disorders and abnormal behaviors in autism children. Hainan Medical Journal. 2021;32(9):1148–50. 10.3969/j.issn.1003-6350.2021.09.016

[29] Jingang Z. Therapeutic effect of acupuncture at Du Mai point combined with interest oriented floor play therapy on children with autism. Capital Medicine. 2021;28(24):148–50. 10.3969/j.issn.1005-8257.2021.24.066

[30] Caixia C, Yongchao L. Application Effect of Acupuncture Combined With Comprehensive Rehabilitation Training on Children With Autism. China Health Standard Management. 2020;11(9):81–4. 10.3969/j.issn.1674-9316.2020.09.034

[31] Wen T, Xiangdong Y. Effect analysis of acupuncture combined with structured education mode in the treatment of children with autism. Journal of Qiqihar Medical College. 2020;41(12):1505–7. 10.3969/j.issn.1002-1256.2020.12.019

[32] Nuo L, Jie-ling L, Zhen-huan L, Yong Z, Bin-xu J, Wen-jie F, et al. Clinical observation on acupuncture at thirteen ghost acupoints for children with autism spectrum disorder. J Acupunct Tuina Sci. 2017;15:344–8. 10.1007/s11726-017-1025-8

[33] Kong F, Hu D, Yuan Q, Zhou W, Li P. Efficacy of acupuncture on children with autism spectrum disorder. Int J Clin Exp Med. 2018;11(12):13775–80.

[34] Yan-li W, Qing S, Hui-chun Z, Li-ye S, Chao G. Clinical Study of Du Xue Dao Qi Needling Method for Autism Spectrum Disorder in Children. Shanghai Journal of Acupuncture and Moxibustion. 2020;39(07):856–60.

[35] Feng G, Ning-xia Z, Ning-bo Z, Wen-tao J, Kai G. Clinical effect of Tiaoshen acupuncture combined with special education and training in treatment of speech disorder in children with autism spectrum disorder. China Journal of Traditional Chinese Medicine and Pharmacy. 2019;34(12):5987–9.

[36] Guo-xiang Z, Xiao-qian Y, Jing-jiang Q. Efficacy Observation of Acupuncture at the Governor Vessel Combined with Rehabilitation Training for Autism. Shanghai Journal of Acupuncture and Moxibustion. 2020;39:1570–5. 10.13460/j.issn.1005-0957.2020.13.1053

[37] Weili D, Wei L, Bingxiang M. Effects of acupuncture intervention on core symptoms in children with autism spectrum disorder. Chinese Journal of Rehabilitation Medicine. 2020;35(05):527–32.

[38] Bing-xu J, Nuo L, Yong Z, Xu-guang Q, Zhen-huan L, Yang Y, et al. Effect of acupoint catgut embedding therapy on joint attention and social communication in children with autism spectrum disorder: a randomized controlled trial. Chinese Acupuncture & Moxibustion. 2020;40(02):162–6. 32100502 10.13703/j.0255-2930.20190214-00054

[39] Wen-Liu Z, Fang L, Zhi-Juan T, Fei-Fei W, Yun S. Effects of Acupoint Catgut Embedding on Cognition and Language Function in Children with Autism Spectrum Disorder Based on the Theory of Regulating Mental with Pivot. Journal of Guangzhou University of Traditional Chinese Medicine. 2021;38(05):954–61.

[40] Wanqiong Z. Clinical observation of electroacupuncture combined with rehabilitation training for children with autism. Guangming Journal of Chinese Medicine. 2022;37(02):300–3.

[41] Ningxia Z, Feng G, Wentao J, Ningbo Z, Bingcang Y, Yani F, et al. Clinical Study of the Intervention on Abnormal Behavior in the Children with Autism Treated with Head Electroacupuncture and Special Education. World Journal of Integrated Traditional and Western Medicine. 2015;10(08):1104–6. 10.13935/j.cnki.sjzx.150822

[42] Wang CN, Liu Y, Wei XH, Li LX. [Effects of electroacupuncture combined with behavior therapy on intelligence and behavior of children of autism]. Zhongguo Zhen Jiu. 2007;27(9):660–2. 17926617

[43] Surapaty IA, Simadibrata C, Rejeki ES, Mangunatmadja I. Laser Acupuncture Effects on Speech and Social Interaction in Patients with Autism Spectrum Disorder. Med Acupunct. 2020;32(5):300–9. doi: 10.1089/acu.2020.1417 33101575 PMC7583335

[44] Wong VC, Chen WX. Randomized controlled trial of electro-acupuncture for autism spectrum disorder. Altern Med Rev. 2010;15(2):136–46. 20806998

[45] Sofronoff K, Attwood T, Hinton S. A randomised controlled trial of a CBT intervention for anxiety in children with Asperger syndrome. J Child Psychol Psyc. 2005;46(11):1152–60. doi: 10.1111/j.1469-7610.2005.00411.x 16238662

[46] Wood JJ, Drahota A, Sze K, Van Dyke M, Decker K, Fujii C, et al. Brief report: effects of cognitive behavioral therapy on parent-reported autism symptoms in school-age children with high-functioning autism. J Autism Dev Disord. 2009;39(11):1608–12. doi: 10.1007/s10803-009-0791-7 19562475 PMC2759867

[47] Storch EA, Arnold EB, Lewin AB, Nadeau JM, Jones AM, De Nadai AS, et al. The effect of cognitive-behavioral therapy versus treatment as usual for anxiety in children with autism spectrum disorders: a randomized, controlled trial. J Am Acad Child Psy. 2013;52(2):132–42. doi: 10.1016/j.jaac.2012.11.007 23357440

[48] Koning C, Magill-Evans J, Volden J, Dick B. Efficacy of cognitive behavior therapy-based social skills intervention for school-aged boys with Autism spectrum disorders. Res Autism Spect Dis. 2013;7(10):1282–90.

[49] Andrews L, Attwood T, Sofronoff K. Increasing the appropriate demonstration of affectionate behavior, in children with Asperger syndrome, high functioning autism, and PDD-NOS: A randomized controlled trial. Res Autism Spect Dis. 2013;7(12):1568–78. 10.1016/j.rasd.2013.09.010

[50] Storch EA, Lewin AB, Collier AB, Arnold E, De Nadai AS, Dane BF, et al. A randomized controlled trial of cognitive-behavioral therapy versus treatment as usual for adolescents with autism spectrum disorders and comorbid anxiety. Depress Anxiety. 2015;32(3):174–81. doi: 10.1002/da.22332 25424398 PMC4346416

[51] Wood JJ, Ehrenreich-May J, Alessandri M, Fujii C, Renno P, Laugeson E, et al. Cognitive behavioral therapy for early adolescents with autism spectrum disorders and clinical anxiety: a randomized, controlled trial. Behav Ther. 2015;46(1):7–19. doi: 10.1016/j.beth.2014.01.002 25526831 PMC4272761

[52] Freitag CM, Jensen K, Elsuni L, Sachse M, Herpertz-Dahlmann B, Schulte-Rüther M, et al. Group-based cognitive behavioural psychotherapy for children and adolescents with ASD: the randomized, multicentre, controlled SOSTA-net trial. J Child Psychol Psyc. 2016;57(5):596–605. doi: 10.1111/jcpp.12509 26715086

[53] Langdon PE, Murphy GH, Shepstone L, Wilson ECF, Fowler D, Heavens D, et al. The People with Asperger syndrome and anxiety disorders (PAsSA) trial: a pilot multicentre, single-blind randomised trial of group cognitive-behavioural therapy. Bjpsych Open. 2016;2(2):179–86. doi: 10.1192/bjpo.bp.115.002527 27703772 PMC4995577

[54] Ireri NW, White SW, Mbwayo AW. Treating Anxiety and Social Deficits in Children with Autism Spectrum Disorder in Two Schools in Nairobi, Kenya. J Autism Dev Disord. 2019;49(8):3309–15. doi: 10.1007/s10803-019-04045-6 31093801

[55] Kilburn TR, Sørensen MJ, Thastum M, Rapee RM, Rask CU, Arendt KB, et al. Group based cognitive behavioural therapy for anxiety in children with autism spectrum disorder: A randomised controlled trial in a general child psychiatric hospital setting. Journal of Autism and Developmental Disorders. 2020;available from: 10.1007/s10803-020-04471-x. 32219638

[56] Dawson G, Rogers S, Munson J, Smith M, Winter J, Greenson J, et al. Randomized, controlled trial of an intervention for toddlers with autism: the Early Start Denver Model. Pediatrics. 2010;125(1):e17–23. doi: 10.1542/peds.2009-0958 19948568 PMC4951085

[57] Rogers SJ, Estes A, Lord C, Vismara L, Winter J, Fitzpatrick A, et al. Effects of a Brief Early Start Denver Model (ESDM)-Based Parent Intervention on Toddlers at Risk for Autism Spectrum Disorders: A Randomized Controlled Trial. J Am Acad Child Psy. 2012;51(10):1052–65. 10.1016/j.jaac.2012.08.003 PMC3487718 23021480

[58] Hong-Hua LI, Chun-Li LI, Di G, Xiu-Yu P, DU Lin, Fei-Yong J. Preliminary application of Early Start Denver Model in children with autism spectrum disorder. Chinese Journal of Contemporary Pediatrics. 2018;20:793–8. 10.7499/j.issn.1008-8830.2018.10.002 30369351 PMC7389043

[59] Shihuan W, Xiaobing Z, Yuanyuan Z, Haitao Z, 陈凯云. Effect of early intervention Denver model on toddlers with autism spectrum disorder. Chinese Journal of Child Health Care. 2021;29(12):1300–3.

[60] Gantman A, Kapp SK, Orenski K, Laugeson EA. Social skills training for young adults with high-functioning autism spectrum disorders: a randomized controlled pilot study. J Autism Dev Disord. 2012;42(6):1094–103. doi: 10.1007/s10803-011-1350-6 21915740

[61] Thomeer ML, Lopata C, Volker MA, Toomey JA, Lee GK, Smerbeck AM, et al. Randomized clinical trial replication of a psychosocial treatment for children with high‐functioning autism spectrum disorders. Psychology in the Schools. 2012;49(10):942–54.

[62] White SW, Ollendick T, Albano AM, Oswald D, Johnson C, Southam-Gerow MA, et al. Randomized controlled trial: Multimodal Anxiety and Social Skill Intervention for adolescents with autism spectrum disorder. J Autism Dev Disord. 2013;43(2):382–94. doi: 10.1007/s10803-012-1577-x 22735897 PMC3494811

[63] Yoo H, Bahn G, Cho I, Kim E, Kim J, Min J, et al. A Randomized Controlled Trial of the Korean Version of the PEERS (R) Parent-Assisted Social Skills Training Program for Teens With ASD. Autism Res. 2014;7(1):145–61. 10.1002/aur.1354 24408892

[64] Laugeson EA, Gantman A, Kapp SK, Orenski K, Ellingsen R. A Randomized Controlled Trial to Improve Social Skills in Young Adults with Autism Spectrum Disorder: The UCLA PEERS (R) Program. J Autism Dev Disord. 2015;45(12SI):3978–89. 10.1007/s10803-015-2504-8 26109247

[65] Adibsereshki N, Nesayan A, Asadi GR, Karimlou M. The Effectiveness of Theory of Mind Training On the Social Skills of Children with High Functioning Autism Spectrum Disorders. Iran J Child Neurol. 2015;9(3):40–9. 26401152 PMC4577697

[66] McVey AJ, Dolan BK, Willar KS, Pleiss S, Karst JS, Casnar CL, et al. A Replication and Extension of the PEERS® for Young Adults Social Skills Intervention: Examining Effects on Social Skills and Social Anxiety in Young Adults with Autism Spectrum Disorder. J Autism Dev Disord. 2016;46(12):3739–54. 10.1007/s10803-016-2911-5 27628940 PMC5310211

[67] Dolan BK, Van Hecke AV, Carson AM, Karst JS, Stevens S, Schohl KA, et al. Brief Report: Assessment of Intervention Effects on In Vivo Peer Interactions in Adolescents with Autism Spectrum Disorder (ASD). J Autism Dev Disord. 2016;46(6):2251–9. doi: 10.1007/s10803-016-2738-0 26886470 PMC5291172

[68] Leaf JB, Leaf JA, Milne C, Taubman M, Oppenheim-Leaf M, Torres N, et al. An Evaluation of a Behaviorally Based Social Skills Group for Individuals Diagnosed with Autism Spectrum Disorder. J Autism Dev Disord. 2017;47(2):243–59. doi: 10.1007/s10803-016-2949-4 27807755

[69] Dekker V, Nauta MH, Timmerman ME, Mulder EJ, van der Veen-Mulders L, van den Hoofdakker BJ, et al. Social skills group training in children with autism spectrum disorder: a randomized controlled trial. Eur Child Adoles Psy. 2019;28(3):415–24. doi: 10.1007/s00787-018-1205-1 30032394 PMC6407743

[70] Vernon TW, Holden AN, Barrett AC, Bradshaw J, Ko JA, McGarry ES, et al. A Pilot Randomized Clinical Trial of an Enhanced Pivotal Response Treatment Approach for Young Children with Autism: The PRISM Model. J Autism Dev Disord. 2019;49(6):2358–73. doi: 10.1007/s10803-019-03909-1 30756274

[71] Kazemi F, Abolghasemi A. Effectiveness of play-based empathy training on social skills in students with autistic spectrum Disorders. Arch Psychiatr Psych. 2019;21(3):71–6. 10.12740/APP/105490

[72] Bauminger-Zviely N, Estrugo Y, Samuel-Magal K, Friedlin A, Heishrik L, Koren D, et al. Communicating Without Words: School-Based RCT Social Intervention in Minimally Verbal Peer Dyads with ASD. J Clin Child Adolesc. 2020;49(6):837–53. doi: 10.1080/15374416.2019.1660985 31560567

[73] Gorenstein M, Giserman-Kiss I, Feldman E, Isenstein EL, Donnelly L, Wang AT, et al. Brief Report: A Job-Based Social Skills Program (JOBSS) for Adults with Autism Spectrum Disorder: A Pilot Randomized Controlled Trial. J Autism Dev Disord. 2020;50(12):4527–34. doi: 10.1007/s10803-020-04482-8 32297122

[74] Barrett AC, Vernon TW, McGarry ES, Holden AN, Bradshaw J, Ko JA, et al. Social responsiveness and language use associated with an enhanced PRT approach for young children with ASD: Results from a pilot RCT of the PRISM model. Res Autism Spect Dis. 2020;71. 10.1016/j.rasd.2019.101497

[75] Chien YL, Tsai WC, Chen WH, Yang CL, Gau SSF, Soong WT, et al. Effectiveness, durability, and clinical correlates of the PEERS social skills intervention in young adults with autism spectrum disorder: the first evidence outside North America. Psychol Med. 2021:1–11. doi: 10.1017/S0033291721002385 34247667

[76] de Korte MWP, van den Berk-Smeekens I, Buitelaar JK, Staal WG, van Dongen-Boomsma M. Pivotal Response Treatment for School-Aged Children and Adolescents with Autism Spectrum Disorder: A Randomized Controlled Trial. J Autism Dev Disord. 2021;51(12):4506–19. doi: 10.1007/s10803-021-04886-0 33559019

[77] Rabin SJ, Laugeson EA, Mor-Snir I, Golan O. An Israeli RCT of PEERS: Intervention Effectiveness and the Predictive Value of Parental Sensitivity. Journal of Clinical Child and Adolescent Psychology: The Official Journal for the Society of Clinical Child and Adolescent Psychology, American Psychological Association, Division 53. 2021;50(6):933–49. 10.1080/15374416.2020.1796681 32780594

[78] Temkin AB, Beaumont R, Wkya K, Hariton JR, Flye BL, Sheridan E, et al. Secret Agent Society: A Randomized Controlled Trial of a Transdiagnostic Youth Social Skills Group Treatment. Res Child Adoles Psy. 2022;available from:10.1007/s10803-020-04471-x. 10.1007/s10803-020-04471-x 35441908

[79] Kovshoff H, Hastings RP, Remington B. Two-Year Outcomes for Children With Autism After the Cessation of Early Intensive Behavioral Intervention. Behav Modif. 2011;35(5):427–50. doi: 10.1177/0145445511405513 21586502

[80] Whitehouse A, Granich J, Alvares G, Busacca M, Cooper MN, Dass A, et al. A randomised controlled trial of an iPad-based application to complement early behavioural intervention in Autism Spectrum Disorder. J Child Psychol Psyc. 2017;58(9):1042–52. doi: 10.1111/jcpp.12752 28543302

[81] Gates JA, Kang E, Lerner MD. Efficacy of group social skills interventions for youth with autism spectrum disorder: A systematic review and meta-analysis. Clin Psychol Rev. 2017;52:164–81. doi: 10.1016/j.cpr.2017.01.006 28130983 PMC5358101

[82] Reichow B, Hume K, Barton EE, Boyd BA. Early intensive behavioral intervention (EIBI) for young children with autism spectrum disorders (ASD). Cochrane Db Syst Rev. 2018;5(5):CD009260. doi: 10.1002/14651858.CD009260.pub3 29742275 PMC6494600

[83] Tiede G, Walton KM. Meta-analysis of naturalistic developmental behavioral interventions for young children with autism spectrum disorder. Autism. 2019;23(8):2080–95. doi: 10.1177/1362361319836371 31018655

[84] Perihan C, Burke M, Bowman-Perrott L, Bicer A, Gallup J, Thompson J, et al. Effects of Cognitive Behavioral Therapy for Reducing Anxiety in Children with High Functioning ASD: A Systematic Review and Meta-Analysis. J Autism Dev Disord. 2020;50(6):1958–72. doi: 10.1007/s10803-019-03949-7 30810842

[85] Darling SJ, Goods M, Ryan NP, Chisholm AK, Haebich K, Payne JM. Behavioral Intervention for Social Challenges in Children and Adolescents: A Systematic Review and Meta-analysis. Jama Pediatr. 2021;175(12):e213982. doi: 10.1001/jamapediatrics.2021.3982 34661613 PMC8524357

