# Supplementary material for: Efficacy of nonpharmacological interventions targeting social function in children and adults with autism spectrum disorder: A systematic review and meta-analysis
Source: PLoS One. 2023 Sep 19;18(9):e0291720. doi: 10.1371/journal.pone.0291720 (PMC10508624; doi:10.1371/journal.pone.0291720)
Supplement: S1 File — (DOCX) [file pone.0291720.s001.docx]

**Efficacy of nonpharmacological interventions targeting social function in children and adults with autism spectrum disorder: a Systematic review and Meta-analysis**

**Supplementary material**

**Content**

[**S1** **Search strategy** 3](#_Toc134819079)

[**S2** **Brief overview** 10](#_Toc134819080)

[**S3** **Risk of bias** 15](#_Toc134819081)

[**S4** **Quality of evidence** 28](#_Toc134819082)

[**S5** **Preferred Reporting Items for Systematic Reviews and Meta-Analyses (PRISMA) checklist** 31](#_Toc134819083)

1. **Search strategy**

**Database:** Medline(via Pubmed)

**Retrieval time:** from inception to April 26^th^ 2022

**Retrieval strategy:**

Acupuncture

(("Autism Spectrum Disorder"[MeSH Terms] OR "Autism Spectrum Disorders"[Title/Abstract] OR "Autistic Spectrum Disorder"[Title/Abstract] OR "Autistic Spectrum Disorders"[Title/Abstract] OR "disorder autistic spectrum"[Title/Abstract] OR "syndrome asperger"[Title/Abstract] OR "Asperger's Disease"[Title/Abstract] OR "Asperger Disease"[Title/Abstract] OR "disease asperger"[Title/Abstract] OR "Asperger Disorder"[Title/Abstract] OR "Asperger Disorders"[Title/Abstract] OR "disorder asperger"[Title/Abstract] OR "disorders asperger"[Title/Abstract] OR "Asperger's Disorder"[Title/Abstract] OR "Aspergers Disorder"[Title/Abstract] OR "disorder asperger s"[Title/Abstract] OR "Asperger's Syndrome"[Title/Abstract] OR "Aspergers Syndrome"[Title/Abstract] OR "syndrome asperger s"[Title/Abstract] OR "disorder autistic"[Title/Abstract] OR "disorders autistic"[Title/Abstract] OR "Kanner's Syndrome"[Title/Abstract] OR "Kanner Syndrome"[Title/Abstract] OR ("autistic disorder"[MeSH Terms] OR ("autistic"[All Fields] AND "disorder"[All Fields]) OR "autistic disorder"[All Fields] OR ("kanners"[All Fields] AND "syndrome"[All Fields]))) AND ("Acupuncture Therapy"[MeSH Terms] OR "Acupuncture Treatment"[Title/Abstract] OR "Acupuncture Treatments"[Title/Abstract] OR "Treatment,Acupuncture"[Title/Abstract] OR "Therapy,Acupuncture"[Title/Abstract] OR "Meridians"[MeSH Terms] OR "Ching Lo"[Title/Abstract] OR "Jing Luo"[Title/Abstract] OR "Jingluo"[Title/Abstract] OR "Acupuncture Point"[Title/Abstract] OR "point acupuncture"[Title/Abstract] OR "points acupuncture"[Title/Abstract] OR "Acupoints"[Title/Abstract] OR "Acupoint"[Title/Abstract] OR "scalp needle"[Title/Abstract] OR "scalp acupuncture"[Title/Abstract] OR "electric acupuncture"[Title/Abstract] OR "electropuncture"[Title/Abstract] OR"intradermal needling"[Title/Abstract] OR "acupoint catgut embedding"[Title/Abstract] OR "elongated needle"[Title/Abstract] OR "catgut embedding"[Title/Abstract] NOT(”pharmacological” [Title/Abstract]OR”medical” [Title/Abstract]))) AND (("randomized controlled trial"[Publication Type] OR "controlled clinical trial"[Publication Type] OR "clinical trials as topic"[MeSH Terms] OR ("randomized"[Title/Abstract] OR "randomised"[Title/Abstract]) OR "randomly"[Title/Abstract] OR "placebo"[Title/Abstract] OR "trial"[Title]) NOT ("animals"[MeSH Terms] NOT "humans"[MeSH Terms]))

Behavioral therapy

(("cognitive behavioral therapy"[MeSH Terms] OR "Behavioral Therapies, Cognitive"[Title/Abstract]OR"Behavioral Therapy, Cognitive"[Title/Abstract]OR"Cognitive Behavioral Therapies"[Title/Abstract]OR"Therapies, Cognitive Behavioral"[Title/Abstract]OR"Therapy, Cognitive Behavioral"[Title/Abstract]OR"Therapy, Cognitive Behavior"[Title/Abstract]OR"Cognitive Behavior Therapy"[Title/Abstract]OR"Cognitive Therapy"[Title/Abstract]OR"Behavior Therapy, Cognitive"[Title/Abstract]OR"Behavior Therapies, Cognitive"[Title/Abstract]OR"Cognitive Behavior Therapies"[Title/Abstract]OR"Therapies, Cognitive Behavior"[Title/Abstract]OR"Cognitive Psychotherapy"[Title/Abstract]OR"Cognitive Psychotherapies"[Title/Abstract]OR"Psychotherapies, Cognitive"[Title/Abstract]OR"Psychotherapy, Cognitive"[Title/Abstract]OR"Therapy, Cognitive"[Title/Abstract]OR"Cognitive Therapies"[Title/Abstract]OR"Therapies, Cognitive"[Title/Abstract]OR"Cognition Therapy"[Title/Abstract]OR"Therapy, Cognition"[Title/Abstract]OR"Cognition Therapies"[Title/Abstract]OR"Therapies, Cognition"[Title/Abstract]OR"Remediation, Cognitive"[Title/Abstract]OR"Denver Model"[Title/Abstract]OR"Remediation, Cognitive"[Title/Abstract]OR"Early intensive behavioral intervention"[Title/Abstract]OR"Early, intervention"[Title/Abstract]OR"Intensive, intervention"[Title/Abstract]OR”social skills”[Title/Abstract] OR”peer interaction”[Title/Abstract] OR”social competence”[Title/Abstract] OR”social functioning”[Title/Abstract] OR”friendship”[Title/Abstract] OR”social interaction”[Title/Abstract] OR”social play”[Title/Abstract] NOT(”pharmacological” [Title/Abstract]OR”medical” [Title/Abstract]))) AND (("randomized controlled trial"[Publication Type] OR "controlled clinical trial"[Publication Type] OR "clinical trials as topic"[MeSH Terms] OR ("randomized"[Title/Abstract] OR "randomised"[Title/Abstract]) OR "randomly"[Title/Abstract] OR "placebo"[Title/Abstract] OR "trial"[Title]) NOT ("animals"[MeSH Terms] NOT "humans"[MeSH Terms]))) AND ("Autism Spectrum Disorder"[MeSH Terms] OR "Autism Spectrum Disorders"[Title/Abstract] OR "Autistic Spectrum Disorder"[Title/Abstract] OR "Autistic Spectrum Disorders"[Title/Abstract] OR "disorder autistic spectrum"[Title/Abstract] OR "syndrome asperger"[Title/Abstract] OR "Asperger's Disease"[Title/Abstract] OR "Asperger Disease"[Title/Abstract] OR "disease asperger"[Title/Abstract] OR "Asperger Disorder"[Title/Abstract] OR "Asperger Disorders"[Title/Abstract] OR "disorder asperger"[Title/Abstract] OR "disorders asperger"[Title/Abstract] OR "Asperger's Disorder"[Title/Abstract] OR "Aspergers Disorder"[Title/Abstract] OR "disorder asperger s"[Title/Abstract] OR "Asperger's Syndrome"[Title/Abstract] OR "Aspergers Syndrome"[Title/Abstract] OR "syndrome asperger s"[Title/Abstract] OR "disorder autistic"[Title/Abstract] OR "disorders autistic"[Title/Abstract] OR "Kanner's Syndrome"[Title/Abstract] OR "Kanner Syndrome"[Title/Abstract] OR ("autistic disorder"[MeSH Terms] OR ("autistic"[All Fields] AND "disorder"[All Fields]) OR "autistic disorder"[All Fields] OR ("kanners"[All Fields] AND "syndrome"[All Fields]

**Database:** Embase

**Retrieval time:**  from inception to April 26^th^ 2022

**Retrieval strategy:**

#1 'acupuncture therapy'/exp OR 'acupuncture therapy' OR 'acupuncture treatment':ab,ti OR 'acupuncture treatments':ab,ti OR 'treatment,acupuncture':ab,ti OR 'therapy,acupuncture':ab,ti OR 'meridians'/exp OR 'ching lo':ab,ti OR 'jing luo':ab,ti OR 'luo, jing':ab,ti OR 'jingluo':ab,ti OR 'acupuncture point':ab,ti OR 'point, acupuncture':ab,ti OR 'points, acupuncture':ab,ti OR 'acupoints':ab,ti OR 'acupoint':ab,ti OR 'scalp needle':ab,ti OR 'scalp acupuncture':ab,ti OR 'electric acupuncture':ab,ti OR 'electropuncture':ab,ti OR 'intradermal needling':ab,ti OR 'acupoint catgut embedding':ab,ti OR 'elongated needle':ab,ti OR 'catgut embedding':ab,ti NOT ('pharmacological':ab,ti OR 'medical':ab,ti)

#2 'cognitive behavior therapy'/exp OR 'behavioral therapies, cognitive':ab,ti OR 'behavioral therapy, cognitive':ab,ti OR 'cognitive behavioral therapies':ab,ti OR 'therapies, cognitive behavioral':ab,ti OR 'therapy, cognitive behavioral':ab,ti OR 'therapy, cognitive behavior':ab,ti OR 'cognitive behavior therapy':ab,ti OR 'cognitive therapy':ab,ti OR 'behavior therapy, cognitive':ab,ti OR 'behavior therapies, cognitive':ab,ti OR 'cognitive behavior therapies':ab,ti OR 'therapies, cognitive behavior':ab,ti OR 'cognitive psychotherapy':ab,ti OR 'cognitive psychotherapies':ab,ti OR 'psychotherapies, cognitive':ab,ti OR 'psychotherapy, cognitive':ab,ti OR 'therapy, cognitive':ab,ti OR 'cognitive therapies':ab,ti OR 'therapies, cognitive':ab,ti OR 'cognition therapy':ab,ti OR 'therapy, cognition':ab,ti OR 'cognition therapies':ab,ti OR 'therapies, cognition':ab,ti OR 'denver model':ab,ti OR 'remediation, cognitive':ab,ti OR 'early intensive behavioral intervention':ab,ti OR 'early, intervention':ab,ti OR 'intensive, intervention':ab,ti OR ‘social skills’:ab,ti OR ‘peer interaction’:ab,ti OR ‘social competence':ab,ti OR ‘social functioning':ab,ti OR ‘friendship':ab,ti OR ‘social interaction':ab,ti OR ‘social play':ab,ti NOT ('pharmacological':ab,ti OR 'medical':ab,ti)

#3 ('randomized controlled trial':it OR 'controlled clinical trial':it OR ('clinical trial'/exp AND topic) OR randomized:ab,ti OR randomised:ab,ti OR randomly:ab,ti OR placebo:ab,ti OR trial:ab,ti) NOT ('animal'/exp NOT 'human'/exp)

#4 'autism spectrum disorder'/exp OR 'autism spectrum disorders':ab,ti OR 'autistic spectrum disorder':ab,ti OR 'autistic spectrum disorders':ab,ti OR 'disorder, autistic spectrum':ab,ti OR 'syndrome, asperger':ab,ti OR 'asperger* disease':ab,ti OR 'asperger* diseases':ab,ti OR 'aspergers disease':ab,ti OR 'disease, asperger*':ab,ti OR 'diseases, asperger*':ab,ti OR 'asperger disease':ab,ti OR 'asperger diseases':ab,ti OR 'disease, asperger':ab,ti OR 'diseases, asperger':ab,ti OR 'asperger disorder':ab,ti OR 'asperger disorders':ab,ti OR 'disorder, asperger':ab,ti OR 'disorders, asperger':ab,ti OR 'asperger* disorder':ab,ti OR 'aspergers disorder':ab,ti OR 'disorder, asperger*':ab,ti OR 'asperger* syndrome':ab,ti OR 'aspergers syndrome':ab,ti OR 'syndrome, asperger*':ab,ti OR 'disorder, autistic':ab,ti OR 'disorders, autistic':ab,ti OR 'kanner* syndrome':ab,ti

Acupuncture

#1 and #3 and #4

Behavioral therapy

#2 and #3 and #4

**Database:** Web of science

**Retrieval time:**  from inception to April 26^th^ 2022

**Retrieval strategy:**

#1 TS=('Acupuncture Therapy' OR 'Acupuncture Treatment' OR 'Acupuncture Treatments' OR 'Treatment,Acupuncture' OR 'Therapy,Acupuncture' OR 'Meridians' OR 'Ching Lo' OR 'Jing Luo' OR 'Luo, Jing' OR 'Jingluo' OR 'Acupuncture Point' OR 'Point, Acupuncture' OR 'Points, Acupuncture' OR 'Acupoints' OR 'Acupoint' OR 'scalp needle' OR 'scalp acupuncture' OR 'intradermal needling' OR 'acupoint catgut embedding' OR 'elongated needle' OR 'catgut embedding' OR 'electric acupuncture' OR 'electropuncture') NOT TS= (‘pharmacological’ OR ‘medical’)

#2 TS= (‘cognitive behavioral therapy’ OR 'Behavioral Therapies, Cognitive' OR 'Behavioral Therapy, Cognitive' OR 'Cognitive Behavioral Therapies' OR 'Therapies, Cognitive Behavioral' OR 'Therapy, Cognitive Behavioral' OR 'Therapy, Cognitive Behavior' OR 'Cognitive Behavior Therapy' OR 'Cognitive Therapy' OR 'Behavior Therapy, Cognitive' OR 'Behavior Therapies, Cognitive' OR 'Cognitive Behavior Therapies' OR 'Therapies, Cognitive Behavior' OR 'Cognitive Psychotherapy' OR 'Cognitive Psychotherapies' OR 'Psychotherapies, Cognitive' OR 'Psychotherapy, Cognitive' OR 'Therapy, Cognitive' OR 'Cognitive Therapies' OR 'Therapies, Cognitive' OR 'Cognition Therapy' OR 'Therapy, Cognition' OR 'Cognition Therapies' OR 'Therapies, Cognition' OR 'Remediation, Cognitive' OR Denver Model OR ‘Remediation, Cognitive‘ OR ‘Early intensive behavioral intervention‘ OR ‘Early, intervention‘ OR ‘Intensive, intervention‘ OR ‘social skills’ OR ‘peer interaction’ OR ‘social competence’ OR ‘social functioning’ OR ‘friendship’ OR ‘social interaction’ OR ‘social play’) NOT TS= (‘pharmacological’ OR ‘medical’)

#3 TS=('randomized controlled trial' OR 'controlled clinical trial' OR 'clinical trials as topic' OR randomized OR randomised OR randomly OR placebo OR trial) NOT TS= ('animals' NOT 'humans')

#4 TS= ('Autism Spectrum Disorder' OR 'Autism Spectrum Disorders' OR 'Autistic Spectrum Disorder' OR 'Autistic Spectrum Disorders' OR 'Disorder, Autistic Spectrum' OR 'Syndrome, Asperger' OR 'Asperger* Disease' OR 'Asperger* Diseases' OR 'Aspergers Disease' OR 'Disease, Asperger*' OR 'Diseases, Asperger*' OR 'Asperger Disease' OR 'Asperger Diseases' OR 'Disease, Asperger' OR 'Diseases, Asperger' OR 'Asperger Disorder' OR 'Asperger Disorders' OR 'Disorder, Asperger' OR 'Disorders, Asperger' OR 'Asperger* Disorder' OR 'Aspergers Disorder' OR 'Disorder, Asperger*' OR 'Asperger* Syndrome' OR 'Aspergers Syndrome' OR 'Syndrome, Asperger*' OR 'Disorder, Autistic' OR 'Disorders, Autistic' OR 'Kanner* Syndrome')

Acupuncture

#1 and #3 and #4

Behavioral therapy

#2 and #3 and #4

**Database:** Psyinfo

**Retrieval time:**  from inception to April 26^th^ 2022

**Retrieval strategy:**

#1 'Acupuncture Therapy' OR 'Acupuncture Treatment' OR 'Acupuncture Treatments' OR 'Treatment,Acupuncture' OR 'Therapy,Acupuncture' OR 'Meridians' OR 'Ching Lo' OR 'Jing Luo' OR 'Luo, Jing' OR 'Jingluo' OR 'Acupuncture Point' OR 'Point, Acupuncture' OR 'Points, Acupuncture' OR 'Acupoints' OR 'Acupoint' OR 'scalp needle' OR 'scalp acupuncture' OR 'intradermal needling' OR 'acupoint catgut embedding' OR 'elongated needle' OR 'catgut embedding' OR 'electric acupuncture' OR 'electropuncture' NOT (‘pharmacological’ OR ‘medical’)

#2 ‘cognitive behavioral therapy’ OR 'Behavioral Therapies, Cognitive' OR 'Behavioral Therapy, Cognitive' OR 'Cognitive Behavioral Therapies' OR 'Therapies, Cognitive Behavioral' OR 'Therapy, Cognitive Behavioral' OR 'Therapy, Cognitive Behavior' OR 'Cognitive Behavior Therapy' OR 'Cognitive Therapy' OR 'Behavior Therapy, Cognitive' OR 'Behavior Therapies, Cognitive' OR 'Cognitive Behavior Therapies' OR 'Therapies, Cognitive Behavior' OR 'Cognitive Psychotherapy' OR 'Cognitive Psychotherapies' OR 'Psychotherapies, Cognitive' OR 'Psychotherapy, Cognitive' OR 'Therapy, Cognitive' OR 'Cognitive Therapies' OR 'Therapies, Cognitive' OR 'Cognition Therapy' OR 'Therapy, Cognition' OR 'Cognition Therapies' OR 'Therapies, Cognition' OR 'Remediation, Cognitive' OR ‘Denver Model’ OR ‘Remediation, Cognitive‘ OR ‘Early intensive behavioral intervention‘ OR ‘Early, intervention‘ OR ‘Intensive, intervention‘ OR ‘social skills’ OR ‘peer interaction’ OR ‘social competence’ OR ‘social functioning’ OR ‘friendship’ OR ‘social interaction’ OR ‘social play’ NOT (‘pharmacological’ OR ‘medical’)

#3 'randomized controlled trial' OR 'controlled clinical trial' OR 'clinical trials as topic' OR randomized OR randomised OR randomly OR placebo OR trial NOT('animals' NOT 'humans')

#4 'Acupuncture Therapy' OR 'Acupuncture Treatment' OR 'Acupuncture Treatments' OR 'Treatment,Acupuncture' OR 'Therapy,Acupuncture' OR 'Meridians' OR 'Ching Lo' OR 'Jing Luo' OR 'Luo, Jing' OR 'Jingluo' OR 'Acupuncture Point' OR 'Point, Acupuncture' OR 'Points, Acupuncture' OR 'Acupoints' OR 'Acupoint' OR 'scalp needle' OR 'scalp acupuncture' OR 'intradermal needling' OR 'acupoint catgut embedding' OR 'elongated needle' OR 'catgut embedding' OR 'electric acupuncture' OR 'electropuncture' NOT (‘pharmacological’ OR ‘medical’)

Acupuncture

#1 and #3 and #4

Behavioral therapy

#2 and #3 and #4

**Database:** CNKI

**Retrieval time:**  from inception to April 26^th^ 2022

**Retrieval strategy:**

Acupuncture

It mainly searches for: (autism spectrum disorder (Mesh term) + autistic disorder (entry term) + asperger*(entry term) + ASD (entry term)) AND (acupuncture (Mesh term) + Catgut Embedment in Acupoint Therapy (Mesh term) + electroacupuncture (Mesh term) + Scalp Acupuncture Therapy (Mesh term)) AND (Randomized Controlled Trials as Topic (Mesh term))

(SU = '自闭症' OR SU = '孤独症' OR SU = '阿斯伯格' OR SU = 'ASD') AND ( SU = '针刺' OR SU = '埋线' OR SU = '电针' OR SU = '头针' NOT SU = '药物') AND (FT = '随机' OR FT = '临床试验' NOT FT = '动物')

Behavioral therapy

It mainly searches for: (autism spectrum disorder (Mesh term) + autistic disorder (entry term) + asperger*(entry term) + ASD (entry term)) AND (Cognitive Behavioral Therapy (Mesh term) + Social skills (Mesh term) +social interaction (entry term) +peer interaction (entry term) +social game (entry term) +friendship (entry term) +early intervention (entry term) +intensive intervention (entry term) +Denver model (entry term)) AND (Randomized Controlled Trials as Topic (Mesh term))

(SU = '自闭症' OR SU = '孤独症' OR SU = '阿斯伯格' OR SU = 'ASD') AND ( SU = '认知行为疗法' OR SU = '丹佛模式' OR SU = '社会技能' OR SU = '同伴交往' OR SU = '社会互动' OR SU = '社交能力' OR SU = '社会性游戏' OR SU = '友谊' OR SU = '早期干预' OR SU = '强化干预' NOT SU = '药物') AND (FT = '随机' OR FT = '临床试验' NOT FT = '动物')

**Database:** Wanfang

**Retrieval time:**  from inception to April 26^th^ 2022

**Retrieval strategy:**

Acupuncture

It mainly searches for: (autism spectrum disorder (Mesh term) + autistic disorder (entry term) + asperger*(entry term) + ASD (entry term)) AND (acupuncture (Mesh term) + Catgut Embedment in Acupoint Therapy (Mesh term) + electroacupuncture (Mesh term) + Scalp Acupuncture Therapy (Mesh term)) AND (Randomized Controlled Trials as Topic (Mesh term))

(自闭症 OR 孤独症 OR 阿斯伯格 OR ASD) AND ((针刺 OR 埋线 OR 电针 OR 头针) NOT 药物) AND ((随机 OR 临床试验) NOT 动物)

Behavioral therapy

It mainly searches for: (autism spectrum disorder (Mesh term) + autistic disorder (entry term) + asperger*(entry term) + ASD (entry term)) AND (Cognitive Behavioral Therapy (Mesh term) + Social skills (Mesh term) +social interaction (entry term) +peer interaction (entry term) +social game (entry term) +friendship (entry term) +early intervention (entry term) +intensive intervention (entry term) +Denver model (entry term)) AND (Randomized Controlled Trials as Topic (Mesh term))

(自闭症 OR 孤独症 OR 阿斯伯格 OR ASD) AND ((认知行为疗法 OR 丹佛模式 OR 社会技能 OR 同伴交往 OR 社会互动 OR 社交能力 OR 社会性游戏 OR 友谊 OR 早期干预 OR 强化干预) NOT 药物) AND ((随机 OR 临床试验) NOT 动物)

1. **Brief overview**

**S2 Table 1** **Brief introductions of scales measuring social outcome indicators**

| **Scales** | **Interpretation** |
| --- | --- |
| Autism Behavior Checklist | The scale is suitable for ASD screening in people aged 3-35. It includes four dimensions sensory, social interaction, physical movement, communication and self-care, with a total of 57 items. The total score of the scale ranged from 0 to 158, proportional to the degree of symptoms and its critical value is 31 and 67 points, meaning positive in the primary screen and ASD highly suspected. |
| Autism Treatment Evaluation Checklist | The scale is suitable for changes in ASD severity in people aged 2-12 It includes five dimensions: language, social interaction, sensory perception and behavior, with a total of 77 items. The total score of the scale ranged from 0 to 179, proportional to the degree of symptoms and its critical value is 50 and 80 points, the boundary between light and moderate as well as moderate and severe. |
| Functional Independence Measure for Children | The scale is suitable for children with developmental disabilities aged 6 months to 21 years. It includes three dimensions: mobility, self-care, and cognition, with a total of 18 items. The scores are from 1 to 7, with 0 meaning complete dependence and 7 meaning total independence. The total score is 126 (18 items x7) and its critical value is 5 points, the boundary between dependence and independence. |
| Childhood Autism Rating Scale | The scale is suitable for ASD screening in people by doctors or children's psychological test professionals. It includes 15 dimensions: interpersonal relationship, imitate, emotion, body movement ability, relationship with inanimate objects, adaption of the environment, visual, auditory, distance feeling, anxiety reaction, language communication, nonverbal communication, activity levels, mental function, the overall impression, with a total of 60 items. The total score of the scale ranged from 0 to 60, proportional to the degree of symptoms and its critical value is 30 points, meaning positive in the primary screen. |
| Pediatric Quality of Life Inventory | The scale is a modular approach to measuring health-related quality of life (HRQOL) in healthy children and adolescents and those with acute and chronic health conditions. It includes four dimensions of physical function, emotional function, social function and school performance, with a total of 21-23 items by age. The score of each dimension is 0 to 20 points. The higher the score, the better the quality of life. |
| Social life ability scale | The scale is suitable for measuring social life ability in people aged 6 months to14 years old. It includes 6 dimensions: self-help, locomotion, occupation, communication, socialization and self-direction, with a total of 132 items. The total score was 0-10, with 0 and 10 indicating poor or normal social life ability respectively. |
| Social Competence with Peers Questionnaire | It was a parent-report questionnaire that consists of 9 statements regarding a child’s social competence with their same aged peers in the past four weeks. The parent rates the extent to which each of the statements is true (ranging from ‘‘Not true’’ to ‘‘Mostly true’’). The higher the score, the better the social competence with peers. |
| Children’s Automatic Thoughts Scale | It is a developmentally sensitive, general measure of negative self-statements across both internalizing and externalizing problems. Four separate subscales of cognitive content are assessed including physical threat, social threat, personal failure, and hostility, with a total of 40 negative self- statements. Using a 5-point Likert scale (0-never to 4-always), children rate how often each thought came to mind over the past week. Thus, the total score of the scale ranges from 0 to 160. |
| Social Responsiveness Scale | It examines interpersonal behavior and communication including social awareness, social cognition, social communication, social motivation, and social mannerisms, with a total of 65 items. Ratings are on a 4-point Likert scale. Higher scores reflect greater impairment. The total score (M = 50; SD = 10), which reflects the severity of social deficits. |
| Spence Child Anxiety Scale | It contains 39 questions relating to situations in which a child could experience feelings of anxiety, and parents indicate on a scale (0–3) how applicable the feelings would be to their child. The scale yields a total score and six subscales, Generalized Anxiety Disorder (GAD), Obsessive Compulsive Disorder (OCD), Specific Phobia (SP), Panic and Agoraphobia (PA), Separation Anxiety Disorder (SAD), Social Anxiety (SA). The score is proportional to the degree of anxiety. |
| Vinland Adaptive Behavior Scale | It is a parent interview that assesses social, communication, motor, and daily living skills. They provide age-equivalent and standard scores through calculating formula for several subscales, including communication, daily living skill, socialization, motor skill and maladaptive behavior domain. The score is proportional to the degree of symptoms. |
| Contextual Assessment of Social Skills | It is a social functioning assessment that was designed primarily for use with adolescents and young adults with high-functioning ASD. It consists of nine items (two items rated using frequency counts and seven items rated on a 7-point Likert scale). Scores below a 6 indicate some level of social skill deficit. |
| Test of Adolescent Social Skills Knowledge | It is a 26-item measure developed for the original PEERS® treatment to assess adolescents’ knowledge of the specific social skills taught during the intervention. Total scores range from 0 to 26. The higher the score, the better the social skills knowledge. |
| Social Skill Questionnaire | The 30-item parent-report version of Spence’s Social Skills Questionnaire (Spence, 1995) assesses a broad range of social skills over the last four weeks. The higher the score, the better the social skills. |
| Social Skills Rating System | The frequency of general social skills in home and school situations is evaluated as reported by parents and teachers with the subscales “Cooperation”, “Assertion”, “Self-control”, and “Responsibility” of the 38-item Social Skills Rating Scale parent version. Ratings are on a 3-point Likert scale. The higher the score, the better the social skills. |

**S2 Table 2 Brief introductions of interventions**

|  | **Intervention** | **Interpretation** |
| --- | --- | --- |
| Acupuncture | Scalp acupuncture | Acupuncture in the head of the specific point line to prevent the diseases of the whole body[1]. The main schools of scalp acupuncture include Lin’s scalp acupuncture and Jiao’s scalp acupuncture. |
|  | Body acupuncture | Generally refers to the acupuncture in various parts of the meridians and acupoints among the body, relative to the scalp acupuncture. The main schools include Jin’s three needle, acupuncture of strengthening brain and so on. |
|  | Catgut embedding | Under the guidance of meridians and collaterals theory of acupuncture, the medical catgut is embedded in the corresponding acupoint area and stimulate the acupoints persistently and gently through a variety of factors, so as to dredge the meridians and collaterals qi and blood to treat diseases[2]. |
|  | Electropuncture | On the basis of needle acupuncture, different low-frequency pulse electric current is introduced into the body through the needle body, so as to achieve the method of treating diseases[1]. |
|  | Laserpuncture | A new method of acupuncture and moxibustion which uses a laser beam to illuminate acupoints to treat diseases. |
| Behavioral therapy | the Cool Kids ASD program | The Cool Kids ASD is a variation of the original Cool Kids Child and Adolescent Anxiety Program[3] and has been developed specifcally for children who have an anxiety disorder comorbid with ASD. The adaptations to the Cool Kids program were made in order to account for the challenges and concrete learning style of children with high functioning autism[4] and included more visual aides, structured worksheets, simplifed cognitive restructuring exercises, relaxation techniques, and concrete exposure tasks. |
|  | SOSTA-FRA | a highly structured, bodyized, cognitive behavioural, group-based social skills training for children and adolescents with HFASD[5]. It combines experimental, operant behavioural and cognitive techniques, computer-based and social learning methods of teaching and practicing social skills. |
|  | MASSI | Designed for adolescents between 12 and 17 years of age, delivered across three modalities: individual therapy, group therapy (social skills training and practice) and family/school involvement[6]. |
|  | BIACA | BIACA includes adaptations to traditional youth CBT protocols to optimize treatment effectiveness. It emphasizes key skills for positive social behavior skills and developmentally appropriate get-togethers that can be used in real-world settings, such as offering others the first turn[7]. |
|  | ESDM | a comprehensive early behavioral intervention for infants to preschool-aged children with ASD that integrates applied behavior analysis (ABA) with developmental and relationship-based approaches. The ESDM was designed to address the needs of toddlers with ASD as young as 12 months. The intervention is provided in a toddler’s natural environment (the home) and is delivered by trained therapists and parents[8]. |
|  | PEERS | A 14-week social skills intervention for adolescents with ASD that targets making and keeping friends. The intervention focuses on small-group instruction of didactic material, role-plays, behavioral rehearsal, coaching, and weekly homework assignments for social skills practice[9]. |
|  | Pivotal Response Treatment | A social skill treatment focused on creating different social communication learning opportunities for the child (depending on individual target goals) and on teaching parents and teachers to implement PRT principles in the natural environment of the child[10]. |
|  | Job Based Social Skills Program | A manualized, 15-week, group-delivered intervention for young adults with ASD that merges job training and social skills intervention and aims to increase the social-pragmatic skills necessary to obtain and maintain employment[11]. |
|  | Secret Agent Society | The program consists of an initial two-hour parent introductory meeting, followed by nine weekly 90-minute child club meetings and concurrent 45–60 min parent group meetings[12]. The program features a multi-level animated computer game that introduces children to skills in recognizing emotions |
|  | Theory of Mind | Intervention programs are established to improve ToM  and social skills in children with ASD. The intervention sessions consisted of:Four sessions of instructions about the emotions; Two sessions were run for the instruction of situational emotions; Three sessions of instructions about desire; Three sessions of instruction about beliefs[13]. |
|  | TOBY | An ASD intervention program that is accessible on a touch-screen device and targets a wide range of developmental abilities. TOBY is not designed to replace individual therapy with clinicians, but rather provide a means for structuring home-based therapy under the guidance of caregivers[14]. |
|  | Play-based empathy training | A program invented by Kazemi, F. for intervening in autism spectrum disorders. The axis of this program is training empathy via playing and narrating social stories[15]. |
|  | S-PSI | A bodyized intervention for children with minimal verbally ASD (MVASD) that aims to enhance peer interaction and social engagement in two core areas that are essential for effective social interaction but noticeably deficient in MVASD—namely, social collaboration and social conversation. Thus, the S-PSI comprises two main curriculum protocols, one for conversation and one for collaboration[16]. |

1. **Risk of bias**


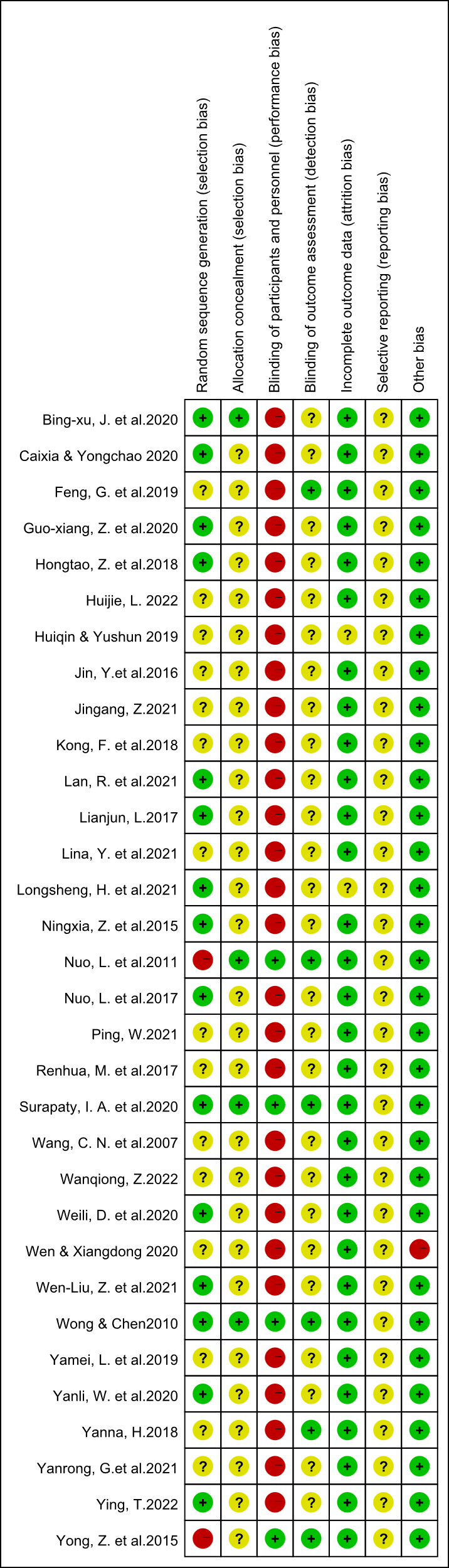

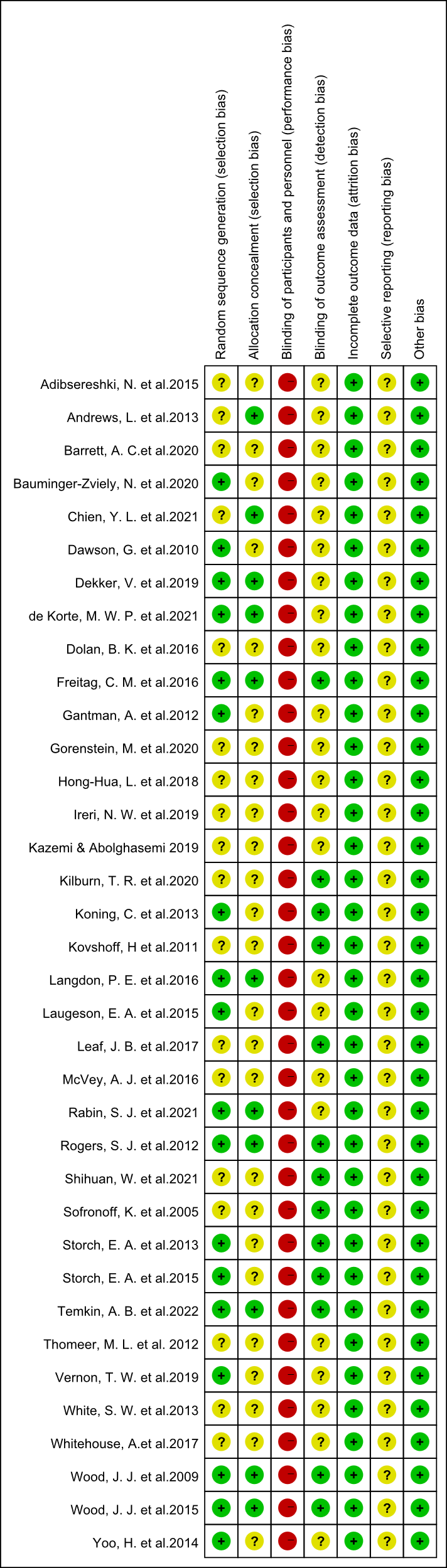


(a) Acupuncture (b) Behavioral therapy

**S3 Figure 1 Risk of bias summary**

a) Acupuncture b) Behavioral therapy


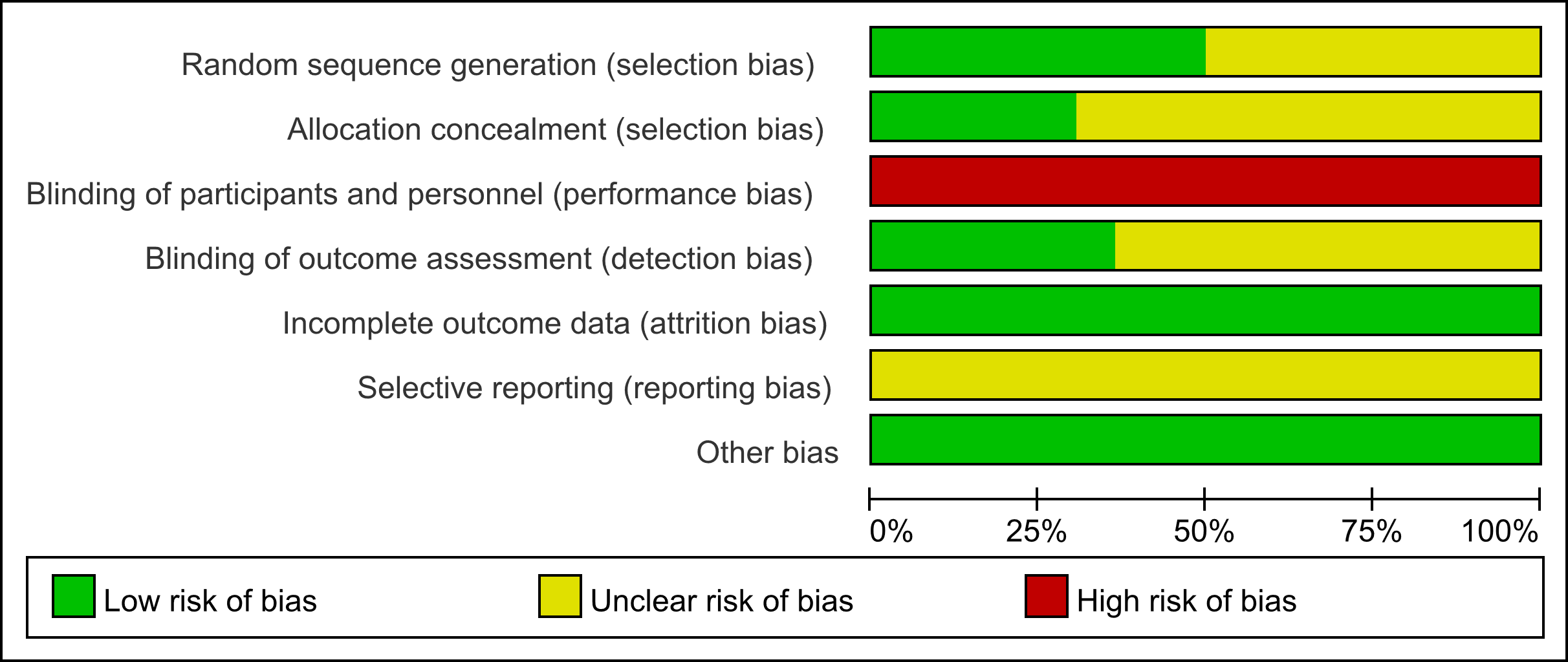

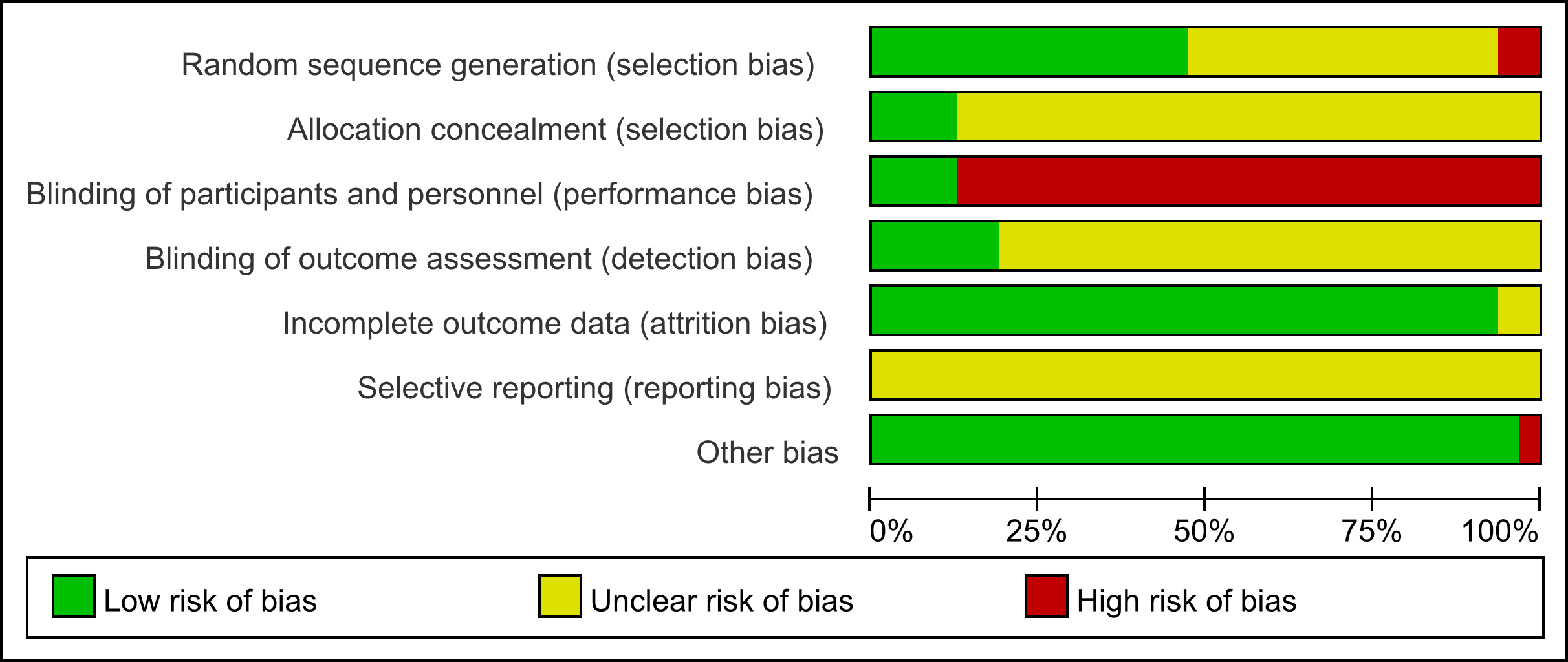


**
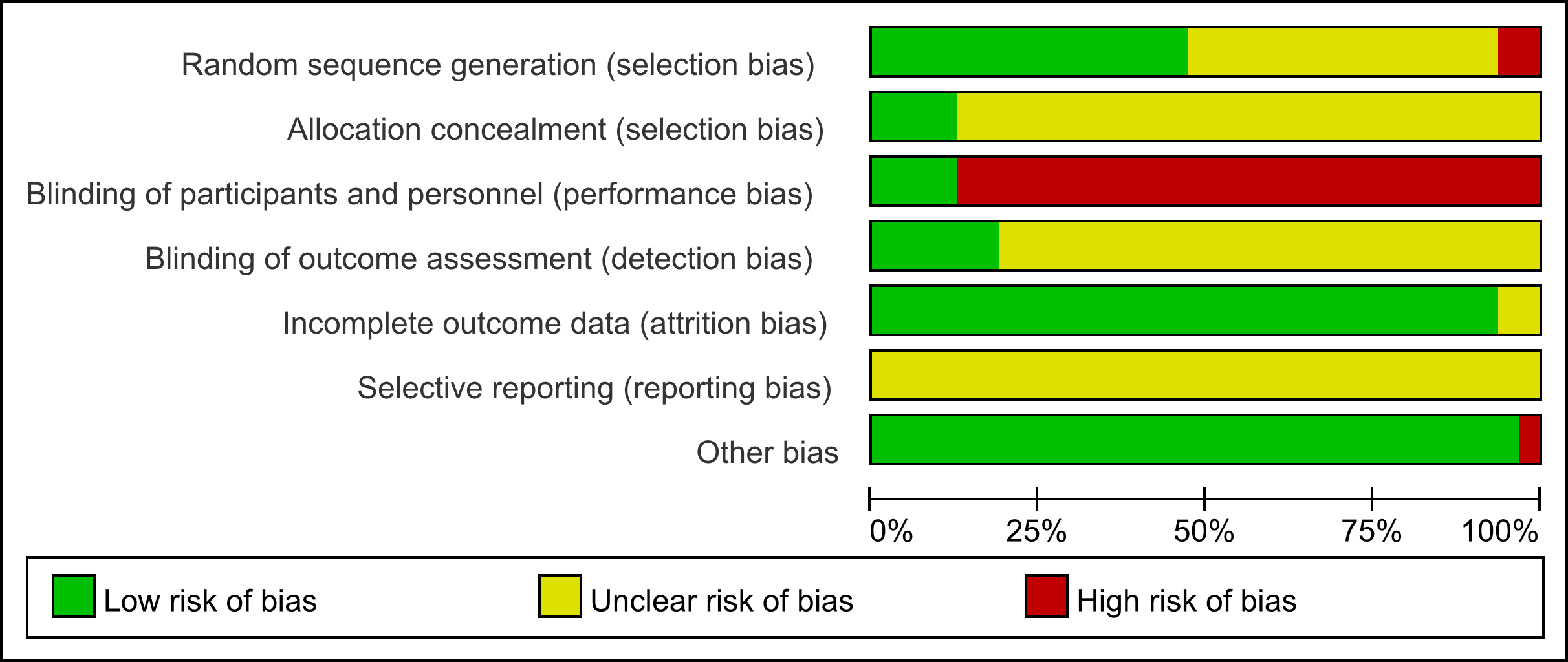
**

**S3 Figure 2 Risk of bias graph**

**S3 Table 1 the source of risk of bias**

| Acupuncture | Overall risk of bias | Behavioral therapy | Overall risk of bias |
| --- | --- | --- | --- |
| Yanrong, G.et al.2021 | Unclear | Andrews, L. et al.2013 | Unclear |
| Huijie, L. 2022 | Unclear | Langdon, P. E. et al.2016 | Low |
| Lianjun, L.2017 | Unclear | Kilburn, T. R. et al.2020 | Unclear |
| Yanna, H.2018 | Unclear | Koning, C. et al.2013 | Low |
| Lina, Y. et al.2021 | Unclear | Sofronoff, K. et al.2005 | Unclear |
| Yamei, L. et al.2019 | Unclear | Freitag,C.M.et al.2016 | Low |
| Ping, W.2021 | Unclear | Ireri, N. W. et al.2019 | Unclear |
| Huiqin & Yushun 2019 | Unclear | Wood, J. J. et al.2015 | Low |
| Jin, Y.et al.2016 | Unclear | Wood, J. J. et al.2009 | Low |
| Renhua, M. et al.2017 | Unclear | Storch, E. A. et al.2015 | Low |
| Hongtao, Z. et al.2018 | Unclear | Storch, E. A. et al.2013 | Low |
| Nuo, L. et al.2017 | Unclear | Dawson, G. et al.2010 | Unclear |
| Kong, F. et al.2018 | Unclear | Hong-Hua, L. et al.2018 | Unclear |
| Feng, G. et al.2019 | Unclear | Rogers, S. J. et al.2012 | Low |
| Yanli, W. et al.2020 | Unclear | Shihuan, W. et al.2021 | Unclear |
| Guo-xiang, Z. et al.2020 | Unclear | Gantman, A. et al.2012 | Unclear |
| Weili, D. et al.2020 | Unclear | Vernon, T. W. et al.2019 | Unclear |
| Ying, T.2022 | Unclear | Leaf, J. B. et al.2017 | Unclear |
| Longsheng, H. et al.2021 | Unclear | McVey, A. J. et al.2016 | Unclear |
| Lan, R. et al.2021 | Unclear | Dolan, B. K. et al.2016 | Unclear |
| Jingang, Z.2021 | Unclear | Thomeer, M. L. et al. 2012 | Unclear |
| Caixia & Yongchao 2020 | Unclear | Yoo, H. et al.2014 | Unclear |
| Wen & Xiangdong 2020 | Unclear | Laugeson, E. A. et al.2015 | Unclear |
| Bing-xu, J. et al.2020 | Low | Temkin, A. B. et al.2022 | Low |
| Wen-Liu, Z. et al.2021 | Unclear | Rabin, S. J. et al.2021 | Low |
| Wong & Chen2010 | Low | White, S. W. et al.2013 | Unclear |
| Wang, C. N. et al.2007 | Unclear | Gorenstein, M. et al.2020 | Unclear |
| Surapaty, I. A. et al.2020 | Low | Kazemi & Abolghasemi 2019 | Unclear |
| Wanqiong,Z.2022 | Unclear | Dekker, V. et al.2019 | Low |
| Ningxia, Z.et al.2015 | Unclear | De Korte, M.W.P.et al.2021 | Low |
| Nuo, L. et al.2011 | High | Chien, Y. L. et al.2021 | Unclear |
| Yong, Z. et al.2015 | High | Bauminger-Zviely, N. et al.2020 | Unclear |
|  |  | Barrett, A. C.et al.2020 | Unclear |
|  |  | Adibsereshki, N. et al.2015 | Unclear |
|  |  | Kovshoff, H et al.2011 | Unclear |
|  |  | Whitehouse, A.et al.2017 | Unclear |

**S3 Figure 3 The assessment criteria for Overall risk of bias**


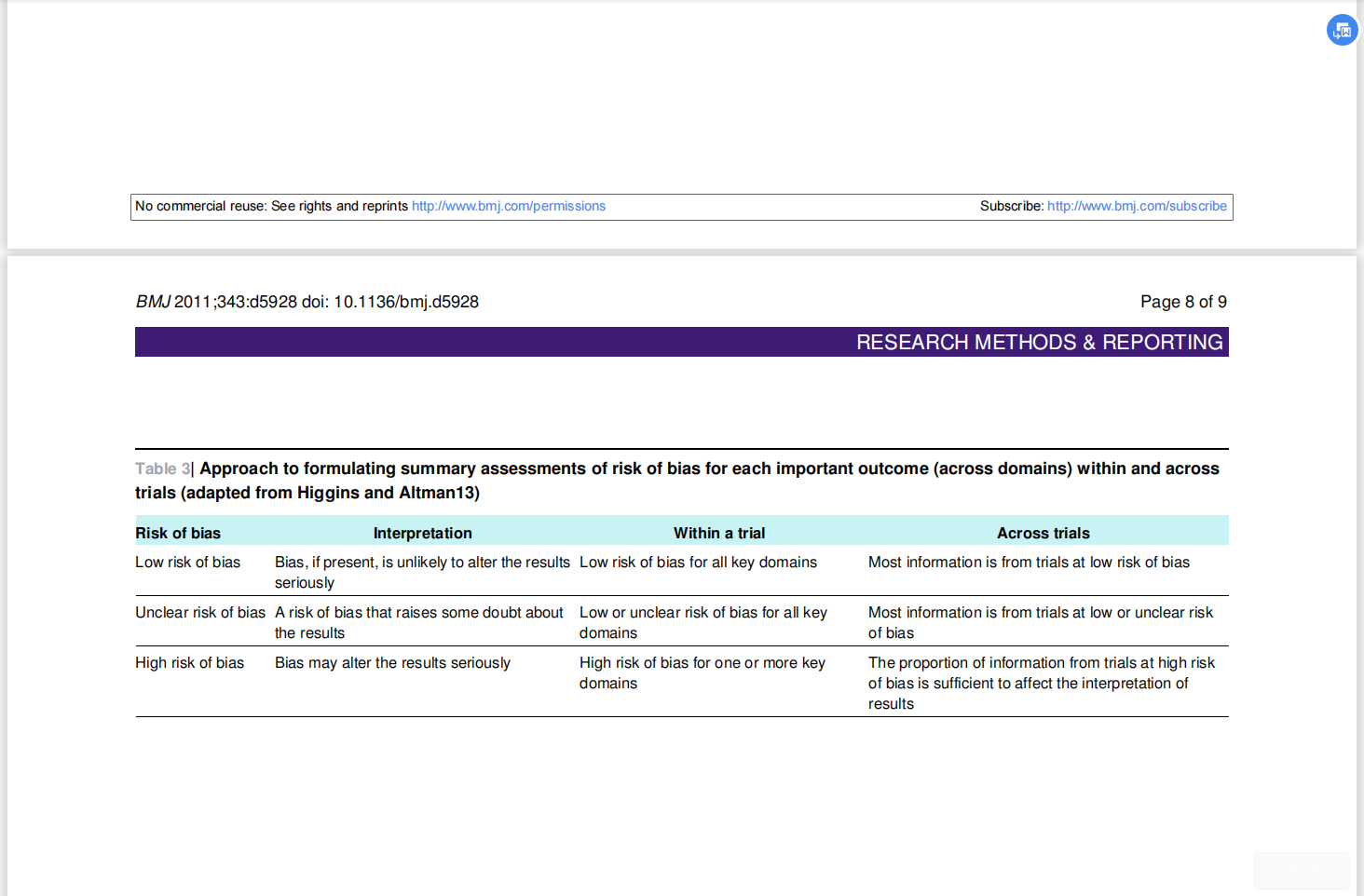


From: Higgins JPT, Altman DG, Gotzsche PC, et al. The Cochrane Collaboration's tool for assessing risk of bias in randomised trials. BMJ 2011;343:d5928

**S3 Table 2 the source of risk of bias**

| Study | Random sequence generation | Allocation concealment | Blinding of participants and personnel | Blinding of outcome assessment | Incomplete outcome data | Selective reporting | Other bias |
| --- | --- | --- | --- | --- | --- | --- | --- |
| Yanrong, G.et al.2021 | No specific method was mentioned | Not mentioned | Hard to blind both sides during acupuncture manipulations and psychotherapy procedures | Not mentioned | Complete outcome data | The original experimental protocols failed to be found | Not found |
| Huijie, L. 2022 | No specific method was mentioned | Not mentioned | Hard to blind both sides during acupuncture manipulations and psychotherapy procedures | Not mentioned | Complete outcome data | The original experimental protocols failed to be found | Not found |
| Lianjun, L.2017 | Random number table | Not mentioned | Hard to blind both sides during acupuncture manipulations and psychotherapy procedures | Not mentioned | Complete outcome data | The original experimental protocols failed to be found | Not found |
| Yanna, H.2018 | No specific method was mentioned | Not mentioned | Hard to blind both sides during acupuncture manipulations and psychotherapy procedures | The outcome was evaluated by isolated medical professional | Complete outcome data | The original experimental protocols failed to be found | Not found |
| Lina, Y. et al.2021 | No specific method was mentioned | Not mentioned | Hard to blind both sides during acupuncture manipulations and psychotherapy procedures | Not mentioned | Complete outcome data | The original experimental protocols failed to be found | Not found |
| Yamei, L. et al.2019 | No specific method was mentioned | Not mentioned | Hard to blind both sides during acupuncture manipulations and psychotherapy procedures | Not mentioned | Complete outcome data | The original experimental protocols failed to be found | Not found |
| Ping, W.2021 | No specific method was mentioned | Not mentioned | Hard to blind both sides during acupuncture manipulations and psychotherapy procedures | Not mentioned | Complete outcome data | The original experimental protocols failed to be found | Not found |
| Huiqin & Yushun 2019 | No specific method was mentioned | Not mentioned | Hard to blind both sides during acupuncture manipulations and psychotherapy procedures | Not mentioned | Complete outcome data | The original experimental protocols failed to be found | Not found |
| Jin, Y.et al.2016 | No specific method was mentioned | Not mentioned | Hard to blind both sides during acupuncture manipulations and psychotherapy procedures | Not mentioned | Complete outcome data | The original experimental protocols failed to be found | Not found |
| Renhua, M. et al.2017 | No specific method was mentioned | Not mentioned | Hard to blind both sides during acupuncture manipulations and psychotherapy procedures | Not mentioned | Complete outcome data | The original experimental protocols failed to be found | Not found |
| Hongtao, Z. et al.2018 | Random number table | Not mentioned | Hard to blind both sides during acupuncture manipulations and psychotherapy procedures | Not mentioned | Complete outcome data | The original experimental protocols failed to be found | Not found |
| Nuo, L. et al.2017 | Random number table | Not mentioned | Hard to blind both sides during acupuncture manipulations and psychotherapy procedures | Not mentioned | Complete outcome data | The original experimental protocols failed to be found | Not found |
| Kong, F. et al.2018 | No specific method was mentioned | Not mentioned | Hard to blind both sides during acupuncture manipulations and psychotherapy procedures | Not mentioned | Complete outcome data | The original experimental protocols failed to be found | Not found |
| Feng, G. et al.2019 | No specific method was mentioned | Not mentioned | Hard to blind both sides during acupuncture manipulations and psychotherapy procedures | The outcome was evaluated by hospital assessment team | Complete outcome data | The original experimental protocols failed to be found | Not found |
| Yanli, W. et al.2020 | Random number table | Not mentioned | Hard to blind both sides during acupuncture manipulations and psychotherapy procedures | Not mentioned | Complete outcome data | The original experimental protocols failed to be found | Not found |
| Guo-xiang, Z. et al.2020 | Random number table | Not mentioned | Hard to blind both sides during acupuncture manipulations and psychotherapy procedures | Not mentioned | Complete outcome data | The original experimental protocols failed to be found | Not found |
| Weili, D. et al.2020 | Random number table | Not mentioned | Hard to blind both sides during acupuncture manipulations and psychotherapy procedures | Not mentioned | Complete outcome data | The original experimental protocols failed to be found | Not found |
| Ying, T.2022 | Random sequence | Not mentioned | Hard to blind both sides during acupuncture manipulations and psychotherapy procedures | Not mentioned | Complete outcome data | The original experimental protocols failed to be found | Not found |
| Longsheng, H. et al.2021 | The simple random grouping method was adopted with rand function of Excel software | Not mentioned | Hard to blind both sides during acupuncture manipulations and psychotherapy procedures | Not mentioned | Complete outcome data | The original experimental protocols failed to be found | Not found |
| Lan, R. et al.2021 | Random number table | Not mentioned | Hard to blind both sides during acupuncture manipulations and psychotherapy procedures | Not mentioned | Complete outcome data | The original experimental protocols failed to be found | Not found |
| Jingang, Z.2021 | No specific method was mentioned | Not mentioned | Hard to blind both sides during acupuncture manipulations and psychotherapy procedures | Not mentioned | Complete outcome data | The original experimental protocols failed to be found | Not found |
| Caixia & Yongchao 2020 | Random number table | Not mentioned | Hard to blind both sides during acupuncture manipulations and psychotherapy procedures | Not mentioned | Complete outcome data | The original experimental protocols failed to be found | Not found |
| Wen & Xiangdong 2020 | No specific method was mentioned | Not mentioned | Hard to blind both sides during acupuncture manipulations and psychotherapy procedures | Not mentioned | Complete outcome data | The original experimental protocols failed to be found | The difference of average age between treatment group and control group was too large. Data error was highly suspected |
| Bing-xu, J. et al.2020 | Random sequence | Used opaque envelope, blinding to the evaluators | Hard to blind both sides during acupuncture manipulations and psychotherapy procedures | Not mentioned | Complete outcome data | The original experimental protocols failed to be found | Not found |
| Wen-Liu, Z. et al.2021 | Random number table | Not mentioned | Hard to blind both sides during acupuncture manipulations and psychotherapy procedures | Not mentioned | Complete outcome data | The original experimental protocols failed to be found | Not found |
| Wong & Chen2010 | Randomized by computer | Allocation was manipulated by the second author | Single-blind by using sham acupuncture | The outcome was evaluated by other people separately | The generation of missing data is unlikely to be related to the true outcome | The original experimental protocols failed to be found | Not found |
| Wang, C. N. et al.2007 | No specific method was mentioned | Not mentioned | Hard to blind both sides during acupuncture manipulations and psychotherapy procedures | Not mentioned | Complete outcome data | The original experimental protocols failed to be found | Not found |
| Surapaty, I. A. et al.2020 | Randomized by computer | The group to which the subject was allocated was unknown by the examiner and parents. | Single-blind by using sham acupuncture | The outcome was evaluated by independent evaluator | Complete outcome data | The original experimental protocols failed to be found | Not found |
| Wanqiong,Z.2022 | No specific method was mentioned | Not mentioned | Hard to blind both sides during acupuncture manipulations and psychotherapy procedures | Not mentioned | Complete outcome data | The original experimental protocols failed to be found | Not found |
| Ningxia, Z.et al.2015 | Participants were numbered according to the registration order, followed by systematic randomization | Not mentioned | Hard to blind both sides during acupuncture manipulations and psychotherapy procedures | Not mentioned | Complete outcome data | The original experimental protocols failed to be found | Not found |
| Nuo, L. et al.2011 | The participants in them were randomized by different hospitals | Not mentioned | Single-blind since participants were separated by different hospitals and treated by independent therapist | The outcome was evaluated by independent psychologist | The generation of missing data is unlikely to be related to the true outcome | The original experimental protocols failed to be found | Not found |
| Yong, Z. et al.2015 | The participants in them were randomized by registration order | Not mentioned | Single-blind since the number and location of acupuncture points were similar between the two groups | The outcome was evaluated by independent psychologist | The generation of missing data is unlikely to be related to the true outcome | The original experimental protocols failed to be found | Not found |
| Andrews, L. et al.2013 | No specific method was mentioned | Random allocation was performed by the first author and parents were informed of their group allocation via telephone | Hard to blind both sides during behavioral therapy procedures | Not mentioned | Complete outcome data | The original experimental protocols failed to be found | Not found |
| Langdon, P. E. et al.2016 | using blocked randomization with random even blocks, stratified by study site | All data were stored independently by the Norwich Clinical Trials | Hard to blind both sides during behavioral therapy procedures | Not mentioned | Complete outcome data | The original experimental protocols failed to be found | Not found |
| Kilburn, T. R. et al.2020 | Randomization was computer generated by a statistical consultant | The therapists and the principal investigator (PI) were blind to sequence list and the allocation until start of treatment | Hard to blind both sides during behavioral therapy procedures | The assessors were blind to trial condition and diagnoses | Complete outcome data | The original experimental protocols failed to be found | Not found |
| Koning, C. et al.2013 | Random number table | Not mentioned | Hard to blind both sides during behavioral therapy procedures | All observational outcome assessments were completed by qualified assessors blind to group | The generation of missing data is unlikely to be related to the true outcome | The original experimental protocols failed to be found | Not found |
| Sofronoff, K. et al.2005 | No specific method was mentioned | Not mentioned | Hard to blind both sides during behavioral therapy procedures | Used independent raters blind to the child’s intervention condition | Complete outcome data | The original experimental protocols failed to be found | Not found |
| Freitag,C.M.et al.2016 | An internet-based, GCP-compliant randomization system was used. | Didn’t blind to patients, families and therapists but not cause significant bias | Hard to blind both sides during behavioral therapy procedures | Teachers who rated outcome measures were masked and blinded. | The generation of missing data is unlikely to be related to the true outcome | The original experimental protocols failed to be found | Not found |
| Ireri, N. W. et al.2019 | No specific method was mentioned | Not mentioned | Hard to blind both sides during behavioral therapy procedures | Not mentioned | The generation of missing data is unlikely to be related to the true outcome | The original experimental protocols failed to be found | Not found |
| Wood, J. J. et al.2015 | Used a computer randomization program | The randomization sequence was concealed from investigators until interventions were assigned | Hard to blind both sides during behavioral therapy procedures | The integrity and caliber of treatment was assessed independently at the University of Miami | The generation of missing data is unlikely to be related to the true outcome | The original experimental protocols failed to be found | Not found |
| Wood, J. J. et al.2009 | Used a computer randomization program | The randomization sequence was concealed from investigators until interventions were assigned | Hard to blind both sides during behavioral therapy procedures | Assessments were conducted by independent evaluators blind to treatment condition | Complete outcome data | The original experimental protocols failed to be found | Not found |
| Storch, E. A. et al.2015 | Used a computer-generated algorithm in a 1:1 ratio | Not mentioned | Hard to blind both sides during behavioral therapy procedures | Clinician-rated measures were administered by trained graduate-level independent evaluators that were blind to intervention condition | The generation of missing data is unlikely to be related to the true outcome | The original experimental protocols failed to be found | Not found |
| Storch, E. A. et al.2013 | Used a computer-generated algorithm in a 1:1 ratio | Not mentioned | Hard to blind both sides during behavioral therapy procedures | Trained independent evaluators who were blind to participants’ intervention condition and not involved in treatment. | The generation of missing data is unlikely to be related to the true outcome | The original experimental protocols failed to be found | Not found |
| Dawson, G. et al.2010 | Used random permuted blocks of 4 | Not mentioned | Hard to blind both sides during behavioral therapy procedures | Not mentioned | The generation of missing data is unlikely to be related to the true outcome | The original experimental protocols failed to be found | Not found |
| Hong-Hua, L. et al.2018 | No specific method was mentioned | Not mentioned | Hard to blind both sides during behavioral therapy procedures | Not mentioned | Complete outcome data | The original experimental protocols failed to be found | Not found |
| Rogers, S. J. et al.2012 | Used a dynamic allocation procedure | Randomized by a statistician in an independent data center | Hard to blind both sides during behavioral therapy procedures | All assessors and data coders were kept unaware of treatment allocation | The generation of missing data is unlikely to be related to the true outcome | The original experimental protocols failed to be found | Not found |
| Shihuan, W. et al.2021 | No specific method was mentioned | Not mentioned | Hard to blind both sides during behavioral therapy procedures | Not mentioned | Complete outcome data | The original experimental protocols failed to be found | Not found |
| Gantman, A. et al.2012 | Randomized by the flip of a coin | Not mentioned | Hard to blind both sides during behavioral therapy procedures | Not mentioned | Complete outcome data | The original experimental protocols failed to be found | Not found |
| Vernon, T. W. et al.2019 | Randomized by the flip of a coin | Not mentioned | Hard to blind both sides during behavioral therapy procedures | Not mentioned | Complete outcome data | The original experimental protocols failed to be found | Not found |
| Leaf, J. B. et al.2017 | No specific method was mentioned | Not mentioned | Hard to blind both sides during behavioral therapy procedures | A blind evaluator measured each participant’s social behavior | Complete outcome data | The original experimental protocols failed to be found | Not found |
| McVey, A. J. et al.2016 | No specific method was mentioned | Not mentioned | Hard to blind both sides during behavioral therapy procedures | Not mentioned | Complete outcome data | The generation of missing data is unlikely to be related to the true outcome | Not found |
| Dolan, B. K. et al.2016 | No specific method was mentioned | Not mentioned | Hard to blind both sides during behavioral therapy procedures | Not mentioned | Complete outcome data | The original experimental protocols failed to be found | Not found |
| Thomeer, M. L. et al. 2012 | No specific method was mentioned | Not mentioned | Hard to blind both sides during behavioral therapy procedures | Not mentioned | Complete outcome data | The original experimental protocols failed to be found | Not found |
| Yoo, H. et al.2014 | The website http://www.random.org was used to randomly assign the eligible teens to the treatment group (TG) or the delayed treatment control group (CG) | Not mentioned | Hard to blind both sides during behavioral therapy procedures | Not mentioned | The generation of missing data is unlikely to be related to the true outcome | The original experimental protocols failed to be found | Not found |
| Laugeson, E. A. et al.2015 | Randomized by the flip of a coin | Not mentioned | Hard to blind both sides during behavioral therapy procedures | Not mentioned | The generation of missing data is unlikely to be related to the true outcome | The original experimental protocols failed to be found | Not found |
| Temkin, A. B. et al.2022 | Randomization was stratifed by clinicsite and done via a computerized random number generator | Not mentioned | Hard to blind both sides during behavioral therapy procedures | The questionnaires were returned via email or regular mail to program facilitators | Complete outcome data | The original experimental protocols failed to be found | Not found |
| Rabin, S. J. et al.2021 | The randomization was conducted by the research coordinator using a computerized program | Group allocation was concealed from the rest of the research team | Hard to blind both sides during behavioral therapy procedures | Not mentioned | Complete outcome data | The original experimental protocols failed to be found | Not found |
| White, S. W. et al.2013 | No specific method was mentioned | Not mentioned | Hard to blind both sides during behavioral therapy procedures | Not mentioned | The generation of missing data is unlikely to be related to the true outcome | The original experimental protocols failed to be found | Not found |
| Gorenstein, M. et al.2020 | No specific method was mentioned | Not mentioned | Hard to blind both sides during behavioral therapy procedures | Not mentioned | Complete outcome data | The original experimental protocols failed to be found | Not found |
| Kazemi & Abolghasemi 2019 | No specific method was mentioned | Not mentioned | Hard to blind both sides during behavioral therapy procedures | Not mentioned | Complete outcome data | The original experimental protocols failed to be found | Not found |
| Dekker, V. et al.2019 | Randomization was done in blocks of five groups per stratum, based on setting, using a computer-generated list of treatment allocations | Performed by research assistants, unaware of the randomization algorithm and unable to access the computer-generated list to conceal the sequence of allocation. | Hard to blind both sides during behavioral therapy procedures | Not mentioned | Complete outcome data | The original experimental protocols failed to be found | Not found |
| De Korte, M.W.P.et al.2021 | A digitalized program (Castor 2019) was used for random sequence generation. | Treatment allocation could not be foreseen by any of the investigators | Hard to blind both sides during behavioral therapy procedures | Not mentioned | Complete outcome data | The original experimental protocols failed to be found | Not found |
| Chien, Y. L. et al.2021 | No specific method was mentioned | Participants were blindly randomized | Hard to blind both sides during behavioral therapy procedures | Not mentioned | Complete outcome data | The original experimental protocols failed to be found | Not found |
| Bauminger-Zviely, N. et al.2020 | Random number table | Not mentioned | Hard to blind both sides during behavioral therapy procedures | Not mentioned | Complete outcome data | The original experimental protocols failed to be found | Not found |
| Barrett, A. C.et al.2020 | No specific method was mentioned | Not mentioned | Hard to blind both sides during behavioral therapy procedures | Not mentioned | Complete outcome data | The original experimental protocols failed to be found | Not found |
| Adibsereshki, N. et al.2015 | No specific method was mentioned | Not mentioned | Hard to blind both sides during behavioral therapy procedures | Not mentioned | Complete outcome data | The original experimental protocols failed to be found | Not found |
| Kovshoff, H et al.2011 | No specific method was mentioned | Not mentioned | Hard to blind both sides during behavioral therapy procedures | The outcome was evaluated independently by a psychometrician | Complete outcome data | The original experimental protocols failed to be found | Not found |
| Whitehouse, A.et al.2017 | No specific method was mentioned | Not mentioned | Hard to blind both sides during behavioral therapy procedures | Not mentioned | The generation of missing data is unlikely to be related to the true outcome | The original experimental protocols failed to be found | Not found |

1. **Quality of evidence**

**S4 Table 1 Evidence quality of non-pharmaceutical therapy**

| **Non-pharmaceutical therapy compared to control for autism spectrum disorder**  **Bibliography:** | | | | | | | | | | | |
| --- | --- | --- | --- | --- | --- | --- | --- | --- | --- | --- | --- |
| **Certainty assessment** | | | | | | | **Summary of findings** | | | | |
| **Participants (studies) Follow-up** | **Risk of bias** | **Inconsistency** | **Indirectness** | **Imprecision** | **Publication bias** | **Overall certainty of evidence** | **Study event rates (%)** | | **Relative effect (95% CI)** | **Anticipated absolute effects** | |
|  |  |  |  |  |  |  | **With control** | **With non-pharmaceutical therapy** |  | **Risk with control** | **Risk difference with non-pharmaceutical therapy** |
| **acupuncture therapy** | | | | | | | | | | | |
| 2228 (30 RCTs) | serious^a^ | serious^b^ | not serious | not serious | publication bias strongly suspected^c^ | ⨁◯◯◯ Very low | 1133 | 1095 | - | - | SMD **0.89 SD higher** (0.71 higher to 1.07 higher) |
| **scalp acupuncture** | | | | | | | | | | | |
| 843 (11 RCTs) | serious^d^ | serious^b^ | not serious | not serious | publication bias strongly suspected^c^ | ⨁◯◯◯ Very low | 420 | 423 | - | - | SMD **0.92 higher** (0.65 higher to 1.18 higher) |
| **body acupuncture** | | | | | | | | | | | |
| 842 (11 RCTs) | serious^e^ | serious^b^ | not serious | not serious | none | ⨁⨁◯◯ Low | 421 | 421 | - | - | SMD **0.76 higher** (0.52 higher to 1.01 higher) |
| **modern acupuncture technology** | | | | | | | | | | | |
| 291 (5 RCTs) | not serious | serious^b^ | not serious | serious^f^ | none | ⨁⨁◯◯ Low | 143 | 148 | - | - | SMD **0.84 higher** (0.32 higher to 1.35 higher) |
| **catgut embedding** | | | | | | | | | | | |
| 120 (2 RCTs) | not serious | not serious | not serious | serious^f^ | none | ⨁⨁⨁◯ Moderate | 60 | 60 | - | - | SMD **0.72 higher** (0.35 higher to 1.09 higher) |
| **Behavioral therapy** | | | | | | | | | | | |
| 1736 (36 RCTs) | not serious | not serious | not serious | not serious | publication bias strongly suspected^c^ | ⨁⨁⨁◯ Moderate | 808 | 928 | - | - | SMD **0.44 SD higher** (0.34 higher to 0.53 higher) |
| **cognitive behavior therapy** | | | | | | | | | | | |
| 610 (11 RCTs) | not serious | not serious | not serious | not serious | none | ⨁⨁⨁⨁ High | 295 | 315 | - | - | SMD **0.42 higher** (0.26 higher to 0.58 higher) |
| **denver model** | | | | | | | | | | | |
| 132 (3 RCTs) | not serious | not serious | not serious | serious^f^ | none | ⨁⨁⨁◯ Moderate | 68 | 64 | - | - | SMD **0.61 higher** (0.23 higher to 0.99 higher) |
| **social skill intervention** | | | | | | | | | | | |
| 784 (19 RCTs) | not serious | not serious | not serious | not serious | publication bias strongly suspected^c^ | ⨁⨁⨁◯ Moderate | 343 | 441 | - | - | SMD **0.56 higher** (0.41 higher to 0.71 higher) |
| **early intensive behavior training** | | | | | | | | | | | |
| 112 (2 RCTs) | serious^g^ | not serious | not serious | serious^f^ | none | ⨁⨁◯◯ Low | 53 | 59 | - |  | MD **0.33 lower** (4.49 lower to 3.82 higher) |
| **Long-term effect (follow-up: range 6 weeks to 2 years)** | | | | | | | | | | | |
| 330 (4 RCTs) | not serious | not serious | not serious | serious^f^ | none | ⨁⨁⨁◯ Moderate | 168 | 162 | - | - | SMD **0.33 higher** (0.12 higher to 0.55 higher) |

**CI:** confidence interval; **MD:** mean difference; **SMD:** standardised mean difference

**Explanations**

a. Most studies fail to mention the allocation concealment and blinding of outcome assessment.

b. Point estimates vary across trials, confidence intervals with minimal overlap and tests for heterogeneity significant

c. The funnel plot of this intervention is asymmetric.

d. Most studies fail to mention the random sequence genertation, allocation concealment and blinding of outcome assessment.

e. Most studies fail to mention the random sequence genertation and blinding of outcome assessment.

f. The sample size does not meet the OIS standard.

g. All studies fail to mention the random sequence genertation and allocation concealment.

1. **Preferred Reporting Items for Systematic Reviews and Meta-Analyses (PRISMA) checklist**

**S5 Table 1 PRISMA Checklist**

| **Section/topic** | **#** | **Checklist item** | **Reported on page #** |
| --- | --- | --- | --- |
| **TITLE** | | |  |
| Title | 1 | Identify the report as a systematic review, meta-analysis, or both. | 1 |
| **ABSTRACT** | | |  |
| Structured summary | 2 | Provide a structured summary including, as applicable: background; objectives; data sources; study eligibility criteria, participants, and interventions; study appraisal and synthesis methods; results; limitations; conclusions and implications of key findings; systematic review registration number. | 2-3 |
| **INTRODUCTION** | | |  |
| Rationale | 3 | Describe the rationale for the review in the context of what is already known. | 4-5 |
| Objectives | 4 | Provide an explicit statement of questions being addressed with reference to participants, interventions, comparisons, outcomes, and study design (PICOS). | 4-5 |
| **METHODS** | | |  |
| Protocol and registration | 5 | Indicate if a review protocol exists, if and where it can be accessed (e.g., Web address), and, if available, provide registration information including registration number. | 5 |
| Eligibility criteria | 6 | Specify study characteristics (e.g., PICOS, length of follow-up) and report characteristics (e.g., years considered, language, publication status) used as criteria for eligibility, giving rationale. | 5-6 |
| Information sources | 7 | Describe all information sources (e.g., databases with dates of coverage, contact with study authors to identify additional studies) in the search and date last searched. | 5-6 |
| Search | 8 | Present full electronic search strategy for at least one database, including any limits used, such that it could be repeated. | 6 |
| Study selection | 9 | State the process for selecting studies (i.e., screening, eligibility, included in systematic review, and, if applicable, included in the meta-analysis). | 7 |
| Data collection process | 10 | Describe method of data extraction from reports (e.g., piloted forms, independently, in duplicate) and any processes for obtaining and confirming data from investigators. | 7-8 |
| Data items | 11 | List and define all variables for which data were sought (e.g., PICOS, funding sources) and any assumptions and simplifications made. | 7-8 |
| Risk of bias in individual studies | 12 | Describe methods used for assessing risk of bias of individual studies (including specification of whether this was done at the study or outcome level), and how this information is to be used in any data synthesis. | 8 |
| Summary measures | 13 | State the principal summary measures (e.g., risk ratio, difference in means). | 8-9 |
| Synthesis of results | 14 | Describe the methods of handling data and combining results of studies, if done, including measures of consistency (e.g., I ) for each meta-analysis. | 8-9 |

| **Section/topic** | **#** | **Checklist item** | **Reported on page #** |
| --- | --- | --- | --- |
| Risk of bias across studies | 15 | Specify any assessment of risk of bias that may affect the cumulative evidence (e.g., publication bias, selective reporting within studies). | 9-10 |
| Additional analyses | 16 | Describe methods of additional analyses (e.g., sensitivity or subgroup analyses, meta-regression), if done, indicating which were pre-specified. | 9-10 |
| **RESULTS** | | |  |
| Study selection | 17 | Give numbers of studies screened, assessed for eligibility, and included in the review, with reasons for exclusions at each stage, ideally with a flow diagram. | 10 |
| Study characteristics | 18 | For each study, present characteristics for which data were extracted (e.g., study size, PICOS, follow-up period) and provide the citations. | 10-18 |
| Risk of bias within studies | 19 | Present data on risk of bias of each study and, if available, any outcome level assessment (see item 12). | 19-25 |
| Results of individual studies | 20 | For all outcomes considered (benefits or harms), present, for each study: (a) simple summary data for each intervention group (b) effect estimates and confidence intervals, ideally with a forest plot. | 19-25 |
| Synthesis of results | 21 | Present results of each meta-analysis done, including confidence intervals and measures of consistency. | 19-25 |
| Risk of bias across studies | 22 | Present results of any assessment of risk of bias across studies (see Item 15). | 19-25 |
| Additional analysis | 23 | Give results of additional analyses, if done (e.g., sensitivity or subgroup analyses, meta-regression [see Item 16]). | 19-25 |
| **DISCUSSION** | | |  |
| Summary of evidence | 24 | Summarize the main findings including the strength of evidence for each main outcome; consider their relevance to key groups (e.g., healthcare providers, users, and policy makers). | 26-30 |
| Limitations | 25 | Discuss limitations at study and outcome level (e.g., risk of bias), and at review-level (e.g., incomplete retrieval of identified research, reporting bias). | 26-30 |
| Conclusions | 26 | Provide a general interpretation of the results in the context of other evidence, and implications for future research. | 31 |
| **FUNDING** | | |  |
| Funding | 27 | Describe sources of funding for the systematic review and other support (e.g., supply of data); role of funders for the systematic review. | NA |

*From:*  Moher D, Liberati A, Tetzlaff J, Altman DG, The PRISMA Group (2009). Preferred Reporting Items for Systematic Reviews and Meta-Analyses: The PRISMA Statement. PLoS Med 6(7): e1000097. doi:10.1371/journal.pmed1000097

**References:**

1. Chinese terms in physical medicine and rehablitation.: Science Press; 2014.

2. Shusen R. Acupoint catgut embedding therapy.: Chinese Medicine Press; 2011.

3. Lyneham HJ, Abbott MJ, Wignall A, Rapee RM. The cool kids child and adolescent anxiety program children's workbook. Sydney, AU: Macquarie University; 2003.

4. Chalfant A, Rapee RM, Carroll L. Treating anxiety disorders in children with high functioning Autism Spectrum Disorders: A controlled trial. Journal of Autism and Developmental. 2007;37(10):1842-57.

5. Cholemkery H, Freitag CM. SOSTA-FRA: Soziales Kompetenztraining fur Kinder und Jugendliche mit Autismus-Spektrum-Storungen.: Weinheim: Beltz; 2014.

6. White SW, Albano A, Johnson C, Kasari C, Ollendick T. Development of a cognitive-behavioural intervention program to treat anxiety and social defcits in teens with high-functioning autism. Clinical Child and Family Psychology. 2010;13:77-90.

7. Wood JJ, Ehrenreich-May J, Alessandri M, Fujii C, Renno P, Laugeson E, et al. Cognitive behavioral therapy for early adolescents with autism spectrum disorders and clinical anxiety: a randomized, controlled trial. Behav Ther. 2015;46(1):7-19. http://doi.org/10.1016/j.beth.2014.01.002

8. Smith M, Rogers S, Dawson G. The Early Start Denver Model: a comprehensive early intervention approach for toddlers with autism. Preschool Education Programs for Children with Autism. 2008:65-101.

9. McVey AJ, Dolan BK, Willar KS, Pleiss S, Karst JS, Casnar CL, et al. A Replication and Extension of the PEERS® for Young Adults Social Skills Intervention: Examining Effects on Social Skills and Social Anxiety in Young Adults with Autism Spectrum Disorder. J Autism Dev Disord. 2016;46(12):3739-54. http://doi.org/10.1007/s10803-016-2911-5

10. Koegel RL, Koegel LK. The PRT pocket guide: Pivotal response treatment for autism spectrum disorders. Baltimore,London: Paul H. Brookes Publishing Co.; 2012.

11. Gorenstein M, Giserman-Kiss I, Feldman E, Isenstein EL, Donnelly L, Wang AT, et al. Brief Report: A Job-Based Social Skills Program (JOBSS) for Adults with Autism Spectrum Disorder: A Pilot Randomized Controlled Trial. J Autism Dev Disord. 2020;50(12):4527-34. http://doi.org/10.1007/s10803-020-04482-8

12. Beaumont R. Secret Agent Society: Solving the mystery of social encounters—facilitator manual. Brisbane, Australia: The Social Skills Training Institute; 2010.

13. Adibsereshki N, Nesayan A, Asadi GR, Karimlou M. The Effectiveness of Theory of Mind Training On the Social Skills of Children with High Functioning Autism Spectrum Disorders. Iran J Child Neurol. 2015;9(3):40-9.

14. Whitehouse A, Granich J, Alvares G, Busacca M, Cooper MN, Dass A, et al. A randomised controlled trial of an iPad-based application to complement early behavioural intervention in Autism Spectrum Disorder. J Child Psychol Psyc. 2017;58(9):1042-52. http://doi.org/10.1111/jcpp.12752

15. Kazemi F, Abolghasemi A. Effectiveness of play-based empathy training on social skills in students with autistic spectrum Disorders. Arch Psychiatr Psych. 2019;21(3):71-6. http://doi.org/10.12740/APP/105490

16. Bauminger-Zviely N, Estrugo Y, Samuel-Magal K, Friedlin A. The school-based peer social intervention (S-PSI).Unpublished protocol. Ramat-Gan, Israel: School of Education, BarIlan University; 2015.
